# Nuclear actin interactome analysis links actin to KAT14 histone acetyl transferase and mRNA splicing

**DOI:** 10.1242/jcs.226852

**Published:** 2019-04-17

**Authors:** Tiina Viita, Salla Kyheröinen, Bina Prajapati, Jori Virtanen, Mikko J. Frilander, Markku Varjosalo, Maria K. Vartiainen

**Affiliations:** 1 Institute of Biotechnology, University of Helsinki, Helsinki 00014, Finland; 2 Helsinki Institute of Life Science, University of Helsinki, Helsinki 00014, Finland; 3 Proteomics Unit, University of Helsinki, Helsinki 00014, Finland

**Keywords:** hATAC, Actin, Nucleus, Transcription, Histone acetyl transferase, Pre-mRNA processing

## Abstract

In addition to its essential functions within the cytoskeleton, actin also localizes to the cell nucleus, where it is linked to many important nuclear processes from gene expression to maintenance of genomic integrity. However, the molecular mechanisms by which actin operates in the nucleus remain poorly understood. Here, we have used two complementary mass spectrometry (MS) techniques, AP-MS and BioID, to identify binding partners for nuclear actin. Common high-confidence interactions highlight the role of actin in chromatin-remodeling complexes and identify the histone-modifying complex human Ada-Two-A-containing (hATAC) as a novel actin-containing nuclear complex. Actin binds directly to the hATAC subunit KAT14, and modulates its histone acetyl transferase activity *in vitro* and in cells. Transient interactions detected through BioID link actin to several steps of transcription as well as to RNA processing. Alterations in nuclear actin levels disturb alternative splicing in minigene assays, likely by affecting the transcription elongation rate. This interactome analysis thus identifies both novel direct binding partners and functional roles for nuclear actin, as well as forms a platform for further mechanistic studies on how actin operates during essential nuclear processes.

This article has an associated First Person interview with the first author of the paper.

## Abstract (precis)

**Highlighted Article:** Nuclear actin interactome analysis reveals novel binding partners and functions for actin in the nucleus, with direct binding and regulation of the KAT14 HAT and functional link to pre-mRNA processing.

## INTRODUCTION

Actin and its importance in the cytoplasm is well known. Actin filaments and their coordinated assembly, regulated by numerous actin-binding proteins (ABPs), are required for cell migration, cytokinesis and membrane dynamics (Le Clainche and Carlier, 2008; Pollard and Cooper, 2009). Although actin is more recognized from its actions in the cytoplasm, it has been known for decades that it is present inside the nucleus where it was suggested to be involved in transcription (Egly et al., 1984). Today, actin is linked to numerous nuclear functions from gene expression to maintenance of genomic integrity (Viita and Vartiainen, 2017).

Development of probes that recognize different forms of actin, as well as improved live imaging techniques, have proven the significance of actin dynamics in the nucleus (Grosse and Vartiainen, 2013). Nowadays, it is firmly established that nuclear actin can polymerize, and even form phalloidin-stainable filaments, in certain situations, such as upon serum stimulation (Baarlink et al., 2013), cell spreading (Plessner et al., 2015), DNA damage responses (Belin et al., 2015; Schrank et al., 2018; Wang et al., 2017) and during certain cell cycle phases (Baarlink et al., 2017). In the cytoplasm, a large number of actin-binding proteins regulate actin dynamics. However, despite the fact that many actin-binding proteins localize to the nucleus (Kumeta et al., 2012), only very few of them have been shown to regulate nuclear actin dynamics. These include cofilin proteins (Baarlink et al., 2017), diaphanous-related formins (mDia1/2) (Baarlink et al., 2013) and formin-2 (FMN2), together with Spire-1/Spire-2 (Belin et al., 2015) and the Arp2/3 complex (Caridi et al., 2018; Schrank et al., 2018). Collectively these studies have demonstrated that, like cytoplasmic actin, nuclear actin dynamics are very tightly regulated.

Actin is linked to many processes that regulate gene expression. Actin, often together with the actin-related proteins (Arps), is a component of many chromatin-remodeling complexes such as SWI/SNF (Zhao et al., 1998), SWR1 (Mizuguchi et al., 2004), Nu4A (Galarneau et al., 2000), Ino80 (Ayala et al., 2018; Eustermann et al., 2018), Tip60 (Ikura et al., 2000) and SRCAP (Cai et al., 2005) complexes. It has been shown that different ATPases of the chromatin-remodeling complexes can bind actin with their helicase-SAINT-associated (HSA) domain (Szerlong et al., 2008). Recent structural work have begun the shed light on the functional relevance of actin in these complexes. For example, in the Ino80 complex actin, Arp4 and Arp8 form a module that is involved in recognizing the extranucleosomal linker DNA (Knoll et al., 2018). Actin can also regulate gene expression by controlling the activity of specific transcription factors. Perhaps the best-characterized example is serum response factor (SRF), which controls the expression of many cytoskeletal genes in response to changes in actin dynamics. The signal from the actin cytoskeleton to SRF is mediated by the transcription coactivator MRTF-A (also known as MAL or MKL1) (Miralles et al., 2003). MRTF-A binds actin monomers through its RPEL domain, which regulates the nuclear localization and activity of MRTF-A in response to actin dynamics (Vartiainen et al., 2007).

Actin has also been linked directly to the transcription process, because it can be co-purified with all three RNA polymerase (Pol) complexes: Pol I (Fomproix and Percipalle, 2004), Pol II (Egly et al., 1984; Smith et al., 1979) and Pol III (Hu et al., 2004). The precise molecular mechanisms by which actin participates in transcription are still unclear, but balanced nucleo-cytoplasmic shuttling of actin is necessary for transcription (Dopie et al., 2012; Sokolova et al., 2018). Availability of nuclear actin monomers seems to be critical here, since polymerization of nuclear actin into stable filaments (Serebryannyy et al., 2016b) or activation of a mechanosensory complex consisting of emerin, non-muscle myosin II and actin (Le et al., 2016) repress transcription. Our recent genome-wide analysis revealed that actin interacts with essentially all transcribed genes. Actin is found, together with Pol II, near the transcription start sites of most genes, as well as on the gene bodies of highly expressed genes (Sokolova et al., 2018). Actin may thus have several functions during the transcription process, and has, in fact, been implicated in pre-initiation complex (PIC) assembly (Hofmann et al., 2004) as well as transcription elongation via the pTEF-β complex (Qi et al., 2011). In addition, actin has also been shown to bind heterogeneous nuclear ribonucleoproteins (hnRNPs) (Obrdlik et al., 2008; Percipalle et al., 2003, 2002), which could indicate a function for actin in mRNA processing.

Beyond gene expression, nuclear actin has also recently been linked to DNA damage responses and DNA replication. Actin dynamics and formin activity are required for initiation of DNA replication through influencing nuclear transport and loading of replication proteins onto chromatin (Parisis et al., 2017). Nuclear actin dynamics are also important in DNA damage responses as reduced nuclear actin increases the number of DNA double-stranded breaks (DSBs) in cells (Belin et al., 2015). Moreover, it seems that DNA damage promotes nuclear actin filament formation (Belin et al., 2015; Wang et al., 2017). These filaments are needed for the DNA damage repair, as their loss leads to a reduced efficiency of DSB clearance. Two recent papers show that the nuclear actin polymerization, mediated by the Arp2/3 complex, is needed for DBS movement in the nucleus (Caridi et al., 2018; Schrank et al., 2018).

It is evident that actin has many important functions within the nucleus. However, the molecular mechanisms by which actin operates during these essential events have remained largely unclear, with one critical aspect being the relatively limited knowledge that we have on the binding partners for nuclear actin. For this reason, we decided to resolve the nuclear actin interactome by using two complementary mass spectrometry (MS)-based techniques, affinity purification (AP)-MS and proximity-dependent biotin identification (BioID)-MS, allowing us to probe both stable and dynamic interactions of nuclear actin. As expected, stable interactions were found for several components of different chromatin-remodeling complexes known to contain actin. The BioID data, on the other hand, links actin to pre-mRNA processing and transcription. Among the hits, we identify a novel direct binding partner for actin in the nucleus, KAT14, which is part of the histone-modifying complex human Ada-Two-A-Containing complex (hATAC). Further functional analysis reveals that actin inhibits the KAT14 histone transferase activity both *in vitro* and in cells. Collectively, our nuclear actin interactome analysis links actin to novel functions within the nucleus, and provides a platform for further mechanistic studies.

## RESULTS

### Two complementary approaches for obtaining the nuclear actin interactome

**Fig. 1.**
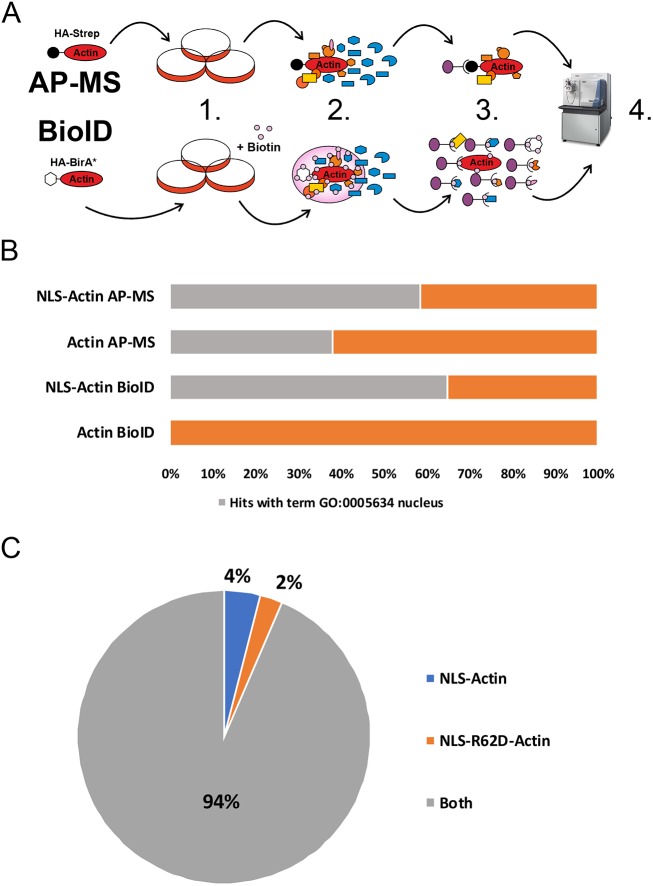
**The nuclear actin interactome resolved by using two different MS approaches and differently localizing actin constructs.** (A) Schematic view of the two different MS approaches, affinity purification MS (AP-MS) and proximity-dependent biotin identification (BioID). (1) Stable, inducible HEK Flp-In cell lines expressing different actin constructs tagged with HA-Strep (a hemagglutinin-conjugated Strep tag) or HA-BirA* (mutated minimal biotin ligase) (2) After induction of actin construct expression, protein complexes are formed with tagged proteins. In BioID, addition of biotin allows HA-BirA* to biotinylate near neighbors of the tagged protein. (3) Single-step purification with StrepTactin to purify formed protein complexes. Milder lysis conditions used in AP-MS to obtain full, intact protein complexes. Purification of biotinylated proteins in BioID-MS enables usage of harsher lysis conditions. (4) Protein digestion to peptides and LC-MS analysis. (B) Fraction of HCIs with the cellular component (CC) GO term ‘nucleus’ (GO:0005634) for the different actin constructs (C) Pie chart showing the fraction of hits that are unique or shared for NLS-actin and NLS-R62D-actin from both AP-MS and BioID. Results in B are from at least two biological replicates from AP-MS and BioID (NLS-actin BioID and NLS-R62D-actin BioID three replicates, rest two replicates). Results in C are from two biological replicates from AP-MS and three biological replicates from BioID.

Actin has been linked to many important nuclear processes from gene expression to maintenance of genomic integrity, but the molecular mechanisms have remained largely unclear. The first step towards elucidating these mechanisms is to identify the binding partners for actin in the nuclear compartment. Here, we have used two complementary MS based approaches, AP-MS (Varjosalo et al., 2013) and BioID-MS (Roux et al., 2012) (Fig. 1A) to identify binding partners for nuclear actin in human embryonic kidney (HEK) cells. These two methods complement each other, because AP-MS aims to catch stable protein complexes in mild lysis conditions, while biotinylation of near neighbors of the bait protein in the BioID technique allows the detection of more transient interactions, and the use of harsher lysis conditions. The low amount of actin in the nucleus compared to cytoplasm, and the ability to distinguish the actual nuclear interactions from cytoplasmic ones (reviewed in Viita and Vartiainen, 2017), make it challenging to study nuclear actin-binding partners. To overcome these obstacles in our study, we have added a nuclear localization signal (NLS) to actin to enrich the amount of actin (see Materials and Methods) in the nucleus. As a control, besides analyzing the diffusively localizing GFP molecule (Fig. S1C), we used actin without the NLS, which allowed us to compare cytoplasmic and nuclear pools of actin with our two MS techniques (Fig. S1A,B; see Materials and Methods for MS data analysis). Primary data from the MS can be found in Table S1 and analyzed data with high-confidence interactions (HCIs) in Table S2. The Database for Annotation, Visualization and Integrated Discovery (DAVID) (Huang et al., 2009) annotation tool revealed that the interactome found with actin with the added NLS contained an increased the amount of proteins with the Gene Ontology (GO) term nucleus (GO:0005634) (58% in NLS-actin AP-MS and 65% in NLS-actin BioID) compared to the interactomes seen without the NLS (38% in actin AP-MS and 0% in actin BioID) (Fig. 1B). This shows that we have been able to enrich nuclear proteins with our NLS-actin constructs. Comparison of nuclear GO terms between AP-MS hits from NLS-actin (before filtering with the data from actin without the NLS) and actin without the NLS revealed very similar nuclear functions (Fig. S2A–C), indicating that addition of the NLS to actin enriches for nuclear proteins, but does not increase unspecific nuclear interactions. We also used a non-polymerizable R62D-actin mutant (Fig. S1A,B; Posern et al., 2002) to study whether the polymerization status of actin affects its nuclear interactions. However, most NLS-actin and NLS-R62D-actin hits were overlapping (Fig. 1C), which indicates that actin does not need the capacity to polymerize to interact with the proteins detected in our experimental setup. For this reason, we combined the actin and R62D-actin datasets and thus overall obtained four different interactomes: NLS-actin AP-MS, NLS-actin BioID, actin AP-MS and actin BioID (Table S2). The shared hits from the actin AP-MS and BioID were, as expected, known regulators of the cytoskeleton and of actin filament assembly (Fig. S3A,B).

### Stable interactions of actin in chromatin-remodeling and -modifying complexes

**Fig. 2.**
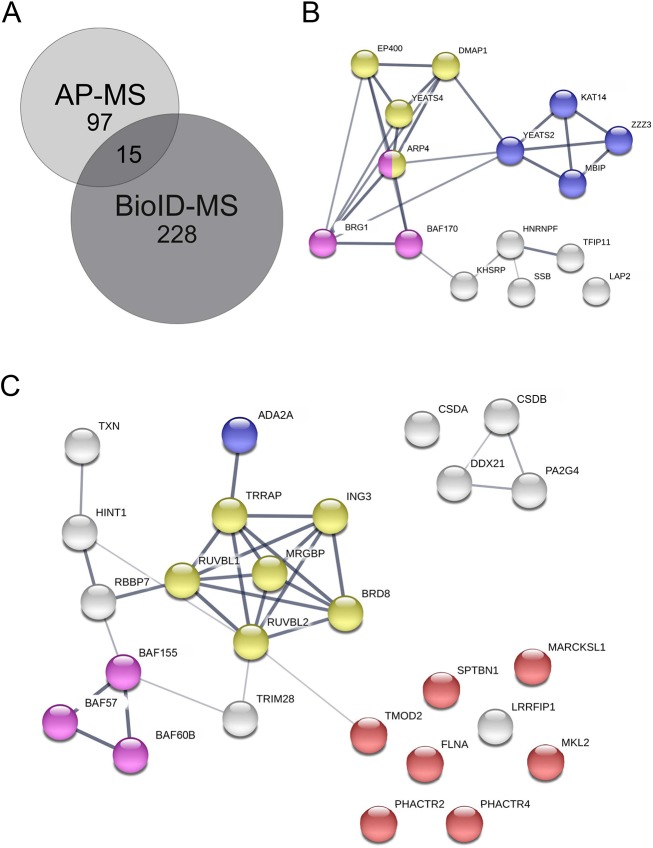
**Shared hits from AP-MS and BioID mainly link actin to chromatin remodeling and modifying complexes.** (A) Number of unique and shared HCIs from AP-MS and BioID experiments for nuclear actin (combined NLS-actin and NLS-R62D-actin). (B) Shared hits from nuclear actin AP-MS and BioID shown as a STRING map. Components of the SWI/SNF complex (GO:0016514) are highlighted in pink, the ATAC complex (GO:0005671) in blue, and the Nu4A histone acetyltransferase complex (GO:0035267) (Nu4A is the core complex of SRCAP/Tip60) in yellow. (C) HCIs for nuclear actin obtained only with the AP-MS technique with SwissProt keywords actin-binding, chromatin remodeling and transcription (26 out of total 96 hits) shown as a STRING map. Different complexes are highlighted as in B, with actin-binding proteins in red.

AP-MS resulted in fewer high-confidence interactions than BioID (Fig. 2A), likely because BioID can also detect more transient interactions. Similar differences between these two approaches have been observed previously for various proteins (Lambert et al., 2015; Liu et al., 2018). Most of the shared hits from the AP-MS and BioID (Fig. 2B) are proteins from chromatin-remodeling complexes, which have been previously established to interact with actin, including SWI/SNF (Zhao et al., 1998) (ARP4, BRG1 and BAF170) and SRCAP/TIP60 (Cai et al., 2005; Ikura et al., 2000) (ARP4, EP400, YEATS4 and DMAP1). Three shared hits, KHSRP (Russo et al., 2011), hnRNPF (Wang and Cambi, 2009) and SSB (Liang et al., 2013), have been linked to alternative splicing. In addition, the shared hits contained several subunits (KAT14, ZZZ3, MBIP and YEATS2) of the hATAC complex (Guelman et al., 2009; Wang et al., 2008) (see also Fig. 5A). To our knowledge, hATAC has not been linked to actin before, although the complex interacts with PCAF, which in turn binds actin (Obrdlik et al., 2008). hATAC complex is a histone acetyl transferase complex, which can acetylate H3 and H4 in mammals (Guelman et al., 2009) and in the fruit fly (Guelman et al., 2006; Suganuma et al., 2008).

The unique hits from the AP-MS contained further subunits from the SWI/SNF (BAF155, BAF57 and BAF60b) (Euskirchen et al., 2012), SRCAP/TIP60 (RUVBL1/2, TRRAP, BRD8, MRGBP and ING3) (Doyon et al., 2004) and hATAC (ADA2A) (Guelman et al., 2009; Wang et al., 2008) complexes (Fig. 2C), which shows that we were able to pull down intact complexes with this technique. In addition, these hits also contained several proteins, which are known to interact with actin, including PHACTR2, PHACTR4 (Allen et al., 2004), FLNA (Shao et al., 2016), TMOD2 (Fowler and Dominguez, 2017), MARCKSL1 (El Amri et al., 2018), SPTBN1 (Machnicka et al., 2014) and MRTF-B (Miralles et al., 2003) (Fig. 2B). Many of these proteins, or their homologs, have also previously been found in the nucleus (Deng et al., 2012; Machnicka et al., 2014; Miralles et al., 2003; Rohrbach et al., 2015; Wiezlak et al., 2012). For instance, MRTF-B is a transcription coactivator of SRF, and presumably regulated by nuclear actin similarly to MRTF-A (Vartiainen et al., 2007). FLNA, an actin-crosslinking protein, interacts with MRTF-A to regulate its activity (Kircher et al., 2015) and has also been linked to DNA repair (Yue et al., 2009), whereas LRRFIP2 is a transcription coactivator (Liu et al., 2005) that interacts with Flightless-I, an actin-binding protein of the gelsolin family (Fong and de Couet, 1999). Finally, over 30% of the unique hits from the AP-MS were linked to translation (Table S2). This may reflect the fact that actin has been linked to both Pol I-mediated transcription and assembly of ribonucleoprotein particles (Percipalle, 2009).

### BioID links actin to transcription and mRNA processing

**Fig. 3.**
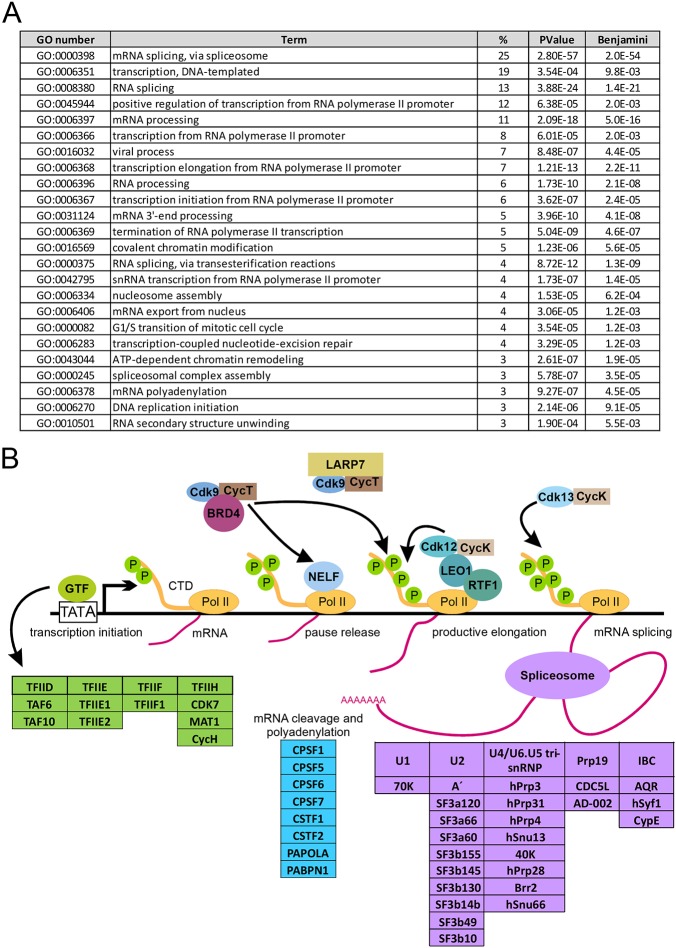
**BioID links actin to mRNA processing and transcription.** (A) DAVID functional annotation chart (cut off Benjamini 10^−3^ and >2% of all hits) with GO Direct Biological Pathways from unique BioID hits. (B) Schematic picture of HCIs from BioID for nuclear actin that are involved in different stages of transcription (Quaresma et al., 2016; Gruber et al., 2014; Wahl and Luhrmann, 2015; Van Oss et al., 2017). Compositions of general transcription factors (GTFs) were obtained from Kyoto Encyclopedia of Genes and Genomes (KEGG) pathway Ko03022 (Kanehisa and Goto, 2000). Note that RNA polymerase II (Pol II) is included for visualization purposes, although it was not a hit in the MS screens for nuclear actin. The phosphate group is indicated with green circle with P and arrows indicate regulatory functions.

The high-confidence interactions from the BioID experiment, link actin to many different functions in the nucleus, such as chromatin remodeling, transcription, DNA replication and mRNA processing (Fig. 3A). Most notably, more than 30% of the unique hits from BioID, as well as a few unique hits from the AP-MS (DDX21 and CSDB), are proteins annotated as being associated with mRNA splicing or processing (Figs 2B and 3A). The hits included protein subunits not only from each of the five spliceosomal small nuclear ribonucleoproteins (snRNPs), but also proteins involved in spliceosome assembly and activation, particularly those associated with complex B or B^act^ formation (Fig. 3B) (Wahl and Lührmann, 2015). These findings imply that actin could be directly associated with pre-mRNA splicing/processing complexes. Alternatively, the co-transcriptional nature of splicing (Herzel et al., 2017), and the involvement of actin in transcription (Fomproix and Percipalle, 2004; Hofmann et al., 2004; Kukalev et al., 2005; Obrdlik et al., 2008; Percipalle et al., 2003; Philimonenko et al., 2004; Sokolova et al., 2018), may explain the detection of the RNA splicing/processing-related proteins.

Indeed, the unique hits from BioID link actin to different stages of transcription from the assembly of the PIC to transcription elongation by Pol II (Fig. 3B). The hits included, for example, subunits of the TFIID, TFIIE, TFIIF and TIIH general transcription factors, supporting the previous data that link actin to PIC formation (Hofmann et al., 2004). In terms of transcription elongation, the pTEF-β complex subunits, CDK9 and cyclin T, which have also previously been linked to monomeric actin (Qi et al., 2011), were hits in our BioID screen (Fig. 3B, Table S2). Interestingly, pTEF-β regulatory proteins LARP7 (BioID), BRD4 (BioID) and DDX21 (AP-MS) (C. Quaresma et al., 2016) were also identified as putative interactors for nuclear actin, indicating that actin could regulate the activity of pTEF-β by modulating its association or release from these factors. Curiously, the BioID hits included also CDK12, CDK13 and cyclin K, which have all been implicated in transcription elongation (Greifenberg et al., 2016). Further experiments are needed to elucidate whether actin generally regulates CDK-mediated Pol II CTD phosphorylation like it has been postulated for the actin-CDK9 interaction (Qi et al., 2011). Of note, none of the RNA polymerase subunits were identified as high-confidence interactions in our assays (Table S2). In agreement with earlier studies (Obrdlik et al., 2008; Percipalle et al., 2002, 2001), we detected interactions between actin and several hnRNP proteins and hnRNP-associated proteins, including hnRNPF (a shared hit), hnRNPM (AP-MS), hnRNPP2 (BioID) and PSF (BioID). Recent studies have linked actin to DNA replication (Parisis et al., 2017), and our BioID screen suggested putative interactions between actin and several replication-linked proteins, including ORC2, ORC5, ORCA, POLA1, POLA2, PRIM1 and PRIM2 (Table S2) (Li and Stillman, 2012; Wu et al., 2014).

**Fig. 4.**
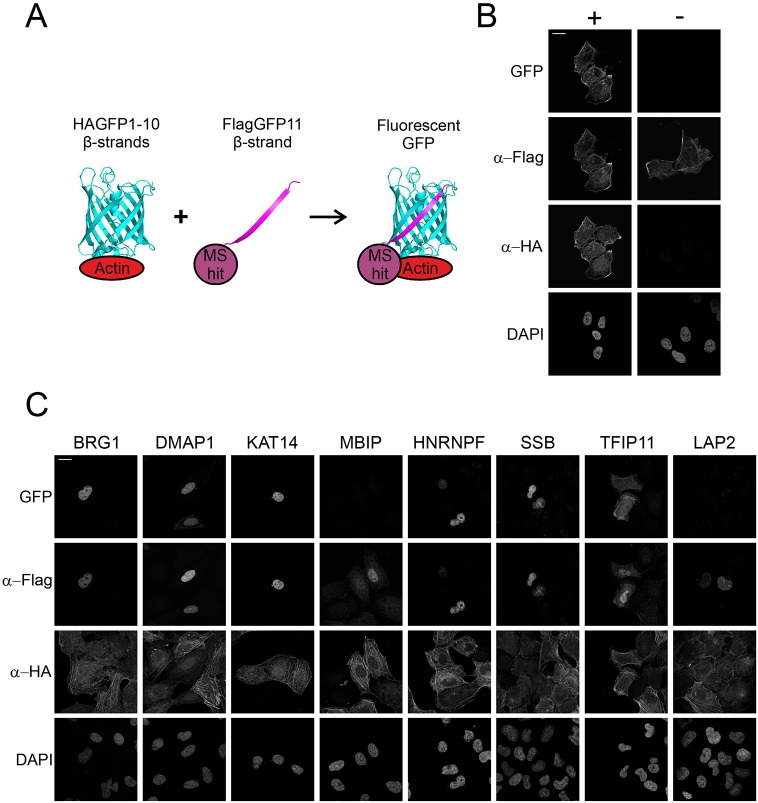
**The BiFC technique validates interactions between shared hits from the AP-MS and BioID interactomes.** (A) Schematic of the BiFC technique. HA–GFP1-10 is attached to bait protein (actin) and Flag–GFP11 is attached to prey protein (MS hit). Fluorescent GFP is reformed if bait and prey interact with each other. Schematic adapted from previous publications (Cabantous et al., 2005; Zhou et al., 2011) and drawn with PyMOL; GFP reference was taken from PDB ID 5B61 (Choi et al., 2017). (B) Confocal microscopy images of the stable inducible U2OS cell line expressing HA–GFP1-10–actin induced (+) and non-induced (−) with tetracycline, transiently transfected with Flag–GFP11–actin and stained with DAPI as well as anti-HA and -Flag antibodies to visualize the expression of the BiFC constructs. The GFP channel shows the BiFC signal, signifying the interaction between the bait and prey protein. (C) BiFC microscopy assay with shared hits from the AP-MS and BioID screens. Confocal microscopy images of tetracycline-induced HA–GFP1-10–actin U2OS cells transiently transfected with the indicated Flag–GFP11 constructs visualized as in B. Scale bars: 20 μm.

To further validate the shared hits from the AP-MS and BioID interactomes, we used the light-microscopy-based bimolecular fluorescence complementation (BiFC) technique (Cabantous et al., 2005; Kerppola, 2008; Zhou et al., 2011), which allowed us to confirm the possible interactions in intact cells (Fig. 4A). For this purpose, we decided to generate a stable, inducible human osteosarcoma (U2OS) cell line expressing HA–GFP1-10–actin and attach the smaller epitope-like tag Flag–GFP11 to the proteins identified as MS hits. To test our method, we used the Flag–GFP11–actin construct together with our inducible HA–GFP1-10–actin cell line and saw reformed GFP (BiFC signal) in transfected cells upon tetracycline induction, but not in non-induced cells (Fig. 4B). Of the eight (BRG1, DMAP1, KAT14, MBIP, hnRNPF, SSB, TFIP11 and LAP2) tested proteins, representing components of different complexes and biological functions of the putative interactors, we were able to verify six by BiFC (Fig. 4C). Only MBIP and LAP2 did not produce detectable GFP fluorescence, despite the fact that both were efficiently expressed in the cells (Fig. 4C). This could indicate that these proteins are either false positives from the MS screens, that the interaction is not direct or that the orientation of the interaction does not allow the reformation of the GFP molecule.

Taken together, by utilizing two complementary MS techniques, we have been able to obtain a global view of the nuclear actin interactome. The identified interactions include already established binding partners for nuclear actin, as well as novel interactions that link actin to its previously described functions, such as chromatin remodeling, DNA replication and transcription. These novel interactions can now be used as the basis for unraveling the molecular mechanisms by which actin operates during these essential nuclear events. In this manuscript, we will further focus on the possible novel functions of actin in the context of the hATAC complex and pre-mRNA splicing.

### Actin binds directly to KAT14 HAT and modulates its HAT activity

**Fig. 5.**
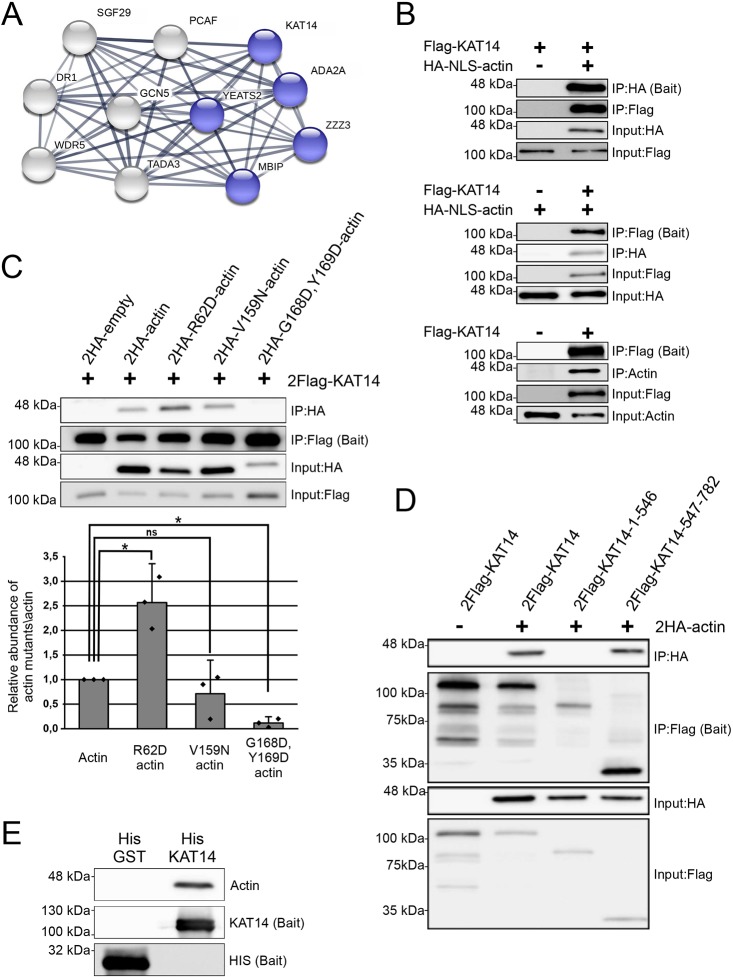
**The hATAC subunit KAT14 directly binds actin and favors binding to actin monomers via its C-terminus.** (A) Schematic of the hATAC complex shown as a STRING map. Proteins indicated in the schematic have been described in Guelman et al. (2009). Proteins highlighted in blue are HCIs in our NLS-actin interactomes. ADA2A was observed only with NLS-actin AP-MS, and the other proteins are shared HCIs from NLS-actin AP-MS and NLS-actin BioID. (B) Western blots of the co-IP assay with 2Flag–KAT14 and 2HA–NLS-actin using HA (upper panel) or Flag beads (middle panel). Actin antibody (AC40) was used to visualize endogenous actin in a co-IP assay with or without overexpressed 2Flag–KAT14 and performed with anti-Flag antibody (lower panel). IP, immunoprecipitation sample. Molecular masses are indicated on the left. (C) Western blots of co-IP assays for 2Flag–KAT14 and the indicated HA-tagged actin mutants (upper panel). The lower panel shows the quantification of the relative amounts of actin mutants co-IP with KAT14. Data is normalized to wild-type actin, and is the mean±s.d. from three independent experiments. Dots in the graph represent individual data points from the independent experiments. *P*-values (*P<0.05) were determined with a one-sided Student's *t*-test showing significance between indicated samples: actin versus R62D-actin (*P*=0.04) and actin versus G168D, Y169D-actin (*P*=0.01). ns, not significant. (D) Western blots of co-IP assay performed for 2HA–actin and indicated 2Flag–KAT14 constructs. (E) Western blots of pulldown assay with actin, His–KAT14 and His–GST detected with the indicated antibodies. Note that for detecting the baits, unequal amounts were loaded on the SDS-PAGE gels (33% of the His–KAT14 sample and less than 1% of His–GST) for visualization purposes. See Fig. S4E for equal loading.

As mentioned above, hATAC is a histone acetyl transferase (HAT) complex that consists of multiple subunits (Fig. 5A) (Guelman et al., 2009; Nagy et al., 2010; Wang et al., 2008). Actin usually interacts with chromatin remodelers with nuclear Arps (reviewed in Kapoor and Shen, 2014), and, quite unexpectedly, the hATAC complex does not contain nuclear Arps. This intrigued us, so we decided to further investigate the relationship between actin and hATAC. The hATAC complex can also interact with PCAF, which is a HAT previously found to associate to actin and hnRNPU (Obrdlik et al., 2008). Interestingly, we did not identify PCAF in our nuclear actin MS analysis (Fig. 5A, Table S2), which might hint that there is another binding partner for actin in the hATAC complex. Here we turned our attention to KAT14, since it produced very strong signal in the BiFC assay (Fig. 4C).

We first verified the interaction between KAT14 and actin through co-immunoprecipitation (co-IP) studies in HeLa cells with overexpressed proteins, utilizing both proteins as baits (Fig. 5B). Additionally, co-IP with overexpressed KAT14 was able to pull down endogenous actin (Fig. 5B). As our interactome data suggested that actin does not require its capacity to polymerize to associate with hATAC (Table S1), we decided to confirm this by co-IP. We did co-IPs with different actin mutants: the non-polymerizable actin mutant (R62D-actin) and a mutant that favors polymerization (V159N-actin) (Posern et al., 2002). In addition, we also tested an actin mutant that has mutations in the hydrophobic cleft (G168D, Y169D-actin) (Cao et al., 2016). Quantified results from the western blots suggest that the mutation in the hydrophobic cleft diminishes the interaction almost completely, and that R62D-actin binds KAT14 more efficiently than wild-type actin (Fig. 5C), further strengthening the notion that KAT14 interacts with actin monomers. We also mapped the binding site for actin in KAT14 by using versions of KAT14 with N- and C-terminal truncations (KAT14-1-546 and KAT14-547-782) (Fig. 5D). Co-IP experiments showed actin binding to the KAT14-547-782 construct, but not to KAT14-1-546, demonstrating that the actin-binding site in KAT14 is in the C-terminus.

**Fig. 6.**
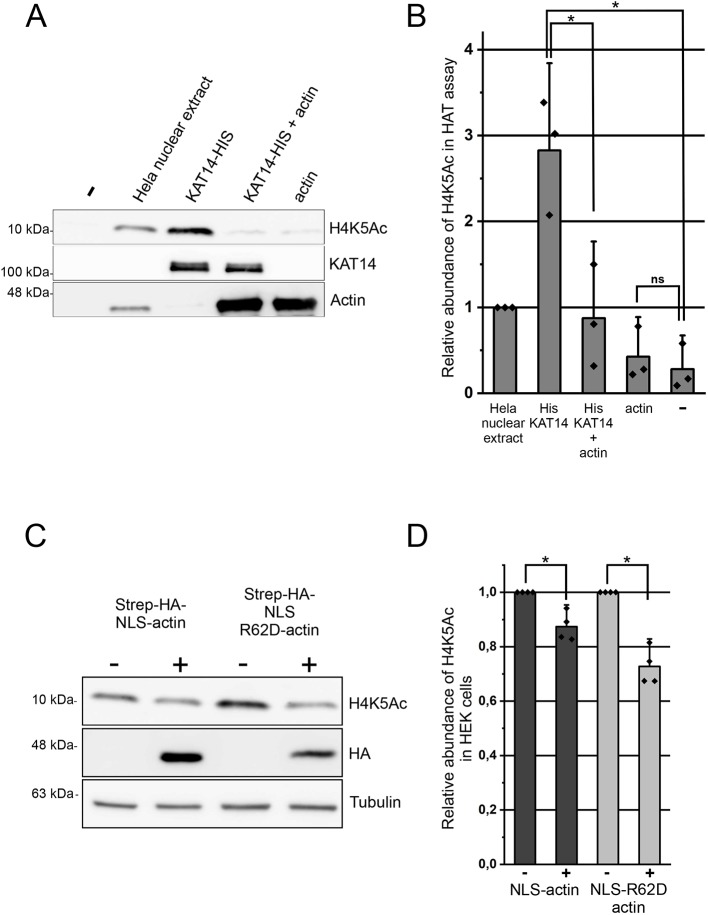
**Actin inhibits KAT14 mediated H4K5 acetylation *in vitro* and in cells.** (A) Western blots of an HAT assay with HeLa nuclear extract, and His-KAT14 without and with addition of actin and actin, probed with the indicated antibodies. –, no extract. (B) Quantification of the relative H4K5Ac abundance from western blots in A. Data is normalized to HeLa nuclear extract and is the mean±s.d. from three independent experiments. Dots in the graph represent individual data points from the independent experiments. *P*-values (*P<0.05) were determined with a two-sided Student's *t*-test showing significance between indicated samples: KAT14 versus KAT14+actin (*P*=0.02) and KAT14 versus negative control (–) (*P*=0.01). ns, not significant. (C) Western blots from total cell lysates of non-induced and tetracycline-induced stable HEK Flp-In cell lines expressing inducible HA-Strep–NLS-actin or HA-Strep–NLS-R62D-actin with the indicated antibodies. (D) Quantification of the relative H4K5Ac abundance from western blots in C. Data is normalized to the non-induced sample, and is mean±s.d. from four independent experiments. Dots in the graph represent individual data points from the independent experiments. *P*-values (*P<0.05) were determined with a one-sided Student's *t*-test showing significance between indicated samples (respectively, *P*=0.04 and *P*=0.01).

To study whether actin binds directly to KAT14, we did a pulldown with purified actin (Fig. S4A) and recombinant His-tagged KAT14 expressed and purified from insect cells (Fig. S4B,C). Actin was detected on beads coated with KAT14, but not those coated with GST (Fig. 5E; Fig. S4E), showing that the interaction between KAT14 and actin is direct. The co-IP experiments with KAT14 truncations suggested that actin would interact with the C-terminus of KAT14. Since this part of KAT14 contains also the HAT activity (Ma et al., 2017), it prompted us to investigate whether actin binding to KAT14 would affect its HAT activity. In experiments with purified proteins, addition of KAT14 to core histones resulted in a marked increase in acetylation of histone 4 lysine 5 (H4K5Ac) (Fig. 6A,B), previously shown to be target for KAT14 (Guelman et al., 2009). Purified actin alone did not increase H4K5Ac above that seen with the negative control (Fig. 6A,B). However, when added together with KAT14, actin significantly decreased the acetylation efficiency of KAT14 (Fig. 6A,B), demonstrating that, *in vitro*, actin negatively influences KAT14 HAT activity. To study whether actin could also regulate histone acetylation in cells, we overexpressed NLS-actin utilizing the inducible Strep-HA–NLS-actin HEK Flp-in cell line used for the AP-MS, and measured H4K5Ac levels. In agreement with the *in vitro* studies, overexpression of NLS-actin led to a significant decrease in H4K5Ac levels (Fig. 6C,D). In line with our MS (Table S1) and co-IP experiments (Fig. 5C), overexpression of also NLS-R62D-actin led to a decrease in H4K5Ac levels (Fig. 6C,D), further supporting the notion that KAT14 seems to prefer binding to monomeric actin.

### Actin influences alternative mRNA splicing regulation

**Fig. 7.**
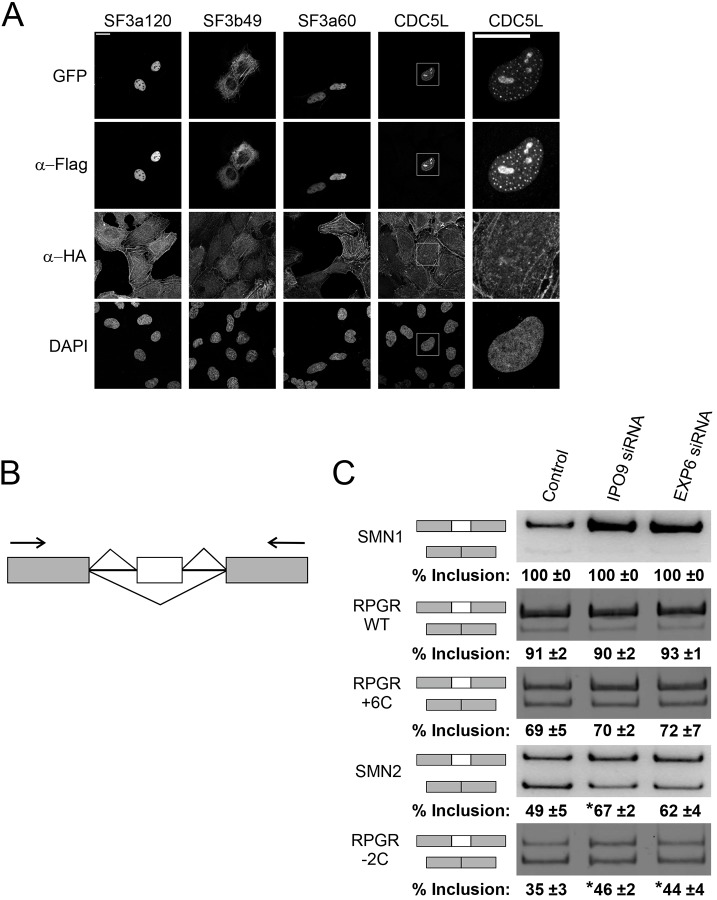
**Actin associates with RNA splicing factors and the disturbance of nuclear actin dynamics affects alternative mRNA splicing of different minigenes.** (A) Confocal microscopy images of a BiFC assay between different splicing factors and actin. Tetracycline-induced HA–GFP1-10–actin U2OS cells transiently transfected with the indicated Flag–GFP11 constructs. Data shown as in Fig. 4B. The right-hand column is a magnified image of CDC5L from the indicated area. Scale bar: 20 μm. (B) Schematic of the minigene assay. The white boxes represent exons and the black lines between the boxes represent introns. Arrows indicate the position of the primers used in the assay. (C) RT-PCR analysis of different minigene splicing from control, Ipo9 and Exp6 siRNA-treated NIH3T3 cells. The order of the used minigenes (SMN1, SMN2, RPGR wt, RPGR −2C and RPGR −6C) is indicated in the figure. The minigene mRNA products are indicated on the left. Quantification from three independent experiments as mean±s.d. percentage of exon inclusion is shown underneath the images. Significant differences between indicated samples are marked with asterisks: SMN2: control siRNA and Ipo9 siRNA (*P*=0.03), RPGR −2C: control siRNA and Ipo9 siRNA (*P*=0.02) and control siRNA and Exp6 siRNA (*P*=0.04).

Previous studies that have mainly focused on the hnRNP proteins (Obrdlik et al., 2008; Percipalle et al., 2002, 2001) have linked actin to pre-mRNA processing, and it has even been suggested that actin would follow the mRNA from the nucleus to the polysomes for translation (reviewed in Percipalle, 2009). However, the functional role for actin in pre-mRNA processing, beyond its role in transcription, has not been demonstrated. Our BioID hits (Fig. 3A,B) further strengthen the link between actin and pre-mRNA processing, but the fact that we also recovered core subunits of the spliceosome suggests that actin could play a role in regulating the splicing process itself. To examine this further, we first utilized the BiFC assay in U2OS cells to confirm selected interactions between actin and splicing factors. Three tested splicing related factors, SF3a120, SF3a60 and CDC5L, gave a fluorescent BiFC signal with actin in the nucleus (Fig. 7A). The BiFC signal between actin and SF3b49 was observed in the cytoplasm, with the tagged SF3b49 also displaying similar subcellular localization in the absence of HA–GFP-1-10–actin expression (data not shown). Intriguingly, the BiFC signal between CDC5L and actin showed a punctuated appearance (Fig. 7A), suggesting that these proteins interact in a specific nuclear compartment. To test the functional significance of nuclear actin for mRNA splicing, we altered nuclear actin levels in mouse fibroblasts (NIH3T3 cells) by means of importin 9 (Ipo9) (Dopie et al., 2012) and exportin 6 (Exp6, also known as XPO6) (Bohnsack et al., 2006; Stuven et al., 2003) depletions, which were confirmed by western blotting (Fig. S5A) or quantitative (q)PCR (Fig. S5B). As demonstrated previously (Dopie et al., 2012), depletion of Ipo9 decreased nuclear actin levels, while depletion of Exp6 increased nuclear actin levels (Fig. S5C,D). Subsequently, we used a set of alternative splicing minigene reporters (Fig. 7B), which each feature different levels of cassette exon inclusion and are sensitive to conditions affecting the splice site choice. Of these, the SMN1 and RPGR wild-type reporters display nearly quantitative cassette exon inclusion levels, while the RPGR +6C, SMN2 and RPGR −2C show progressively reduced exon inclusion levels due to mutations in the 5′ splice site downstream of the cassette exon (Fig. 7C, control lanes) (Roca et al., 2012; Roca and Krainer, 2009). Interestingly, depletion of either Ipo9 or Exp6 increased the cassette exon inclusion specifically in the RPGR −2C and SMN2 reporters, which displayed the lowest inclusion levels in the control knockdown. In contrast, reporters showing high exon inclusion levels in the control knockdown (SMN1, RPGR wt, RPGR +6C) were insensitive to nuclear actin level manipulation (Fig. 7C). This result suggests that alterations in nuclear actin dynamics can influence, either directly or indirectly, alternative splice site choices.

## DISCUSSION

Despite its essential functions in the cell nucleus, our understanding of how actin operates in this cellular compartment at the molecular level is still relatively poor. In the cytoplasm, actin has hundreds of binding partners, and many of these interactions have been characterized in molecular, and even structural, detail. As a step towards understanding the mechanisms by which actin operates in the nucleus, we have here aimed to identify its nuclear binding partners by using two complementary MS methods, AP-MS and BioID. Our analysis identifies previously established binding partners for actin, such as components of chromatin-remodeling complexes, and provides new insights into known actin-regulated processes, such as transcription. In addition, we identify completely novel binding partners and functions for actin in the nucleus.

The abundant cytoplasmic actin pool, as well as biochemical properties of actin itself, pose significant challenges for identifying nucleus-specific interactions for this protein. Cell fractionation is a common approach to study compartment-specific properties of proteins. Based on our own experiences, actin is quite hard to fractionate reliably, and the abundant cytosolic actin contaminates the much less-abundant nuclear fraction easily. In addition, actin polymerization under physiological salt conditions further complicates the issue. Here, we applied two approaches to overcome these issues: (1) increasing the actin amounts in the nucleus by fusing actin with an NLS, and (2) comparing the interactomes to actin without the NLS. Indeed, GO term analysis revealed that our approach led to the identification of an increased number of interacting proteins with the GO term nucleus (GO: 0005634; Fig. 1B), but our approach has also its limitations. One limitation is that we are utilizing tagged actin for both AP-MS and BioID. In the case of AP-MS the tag is equivalent to an epitope tag (less than 4 kDa), but the mutated biotin ligase (BirA*) used in BioID is ∼39 kDa. It is a valid concern that the tag may prevent some interactions of actin, and thus render them undetectable with the approach taken here. For example, although components of the SWI/SNF and SRCAP/TIP60 chromatin-remodeling complexes were readily detected in our nuclear actin interactomes (Fig. 2B,C), no specific subunits of the human INO80 complex scored as high-confidence interactions in our MS screens (Table S2). Earlier studies with yeast INO80 (Kapoor et al., 2013; Shen et al., 2003) and *in vitro* biochemical studies with actin, Arp4 and Arp8 (Fenn et al., 2011) clearly establish the presence of actin in this complex. Recent structural work with the Arp8 module, which also contains actin and Arp4 (Knoll et al., 2018) may explain this discrepancy. In INO80, actin is sandwiched between Arp4 and Arp8, while in the SWI/SNF and SRCAP/Tip60 complexes, actin pairs only with Arp4. Tagging actin could therefore either directly inhibit binding to the INO80 or alternatively, actin could integrate more stably into the INO80 complex than into the other actin-containing remodelers, and, thus, the tagged-actin cannot compete the endogenous actin out of the complex. Development of methods that will allow studies on the endogenous nuclear actin interactions are therefore needed in the future to overcome these limitations. Related to this, our approach also leads to a substantial increase in nuclear actin levels. This could result in false positive interactions, although GO term analysis indicated a significant overlap in the AP-MS interactomes seen for actin with and without the NLS (Fig. S2). Another limitation of our experimental set up relates to the use of the wild-type actin without the NLS to eliminate the cytoplasmic interactions from the nuclear interactomes. As we have shown before, actin shuttles in and out of the nucleus (Dopie et al., 2012), and hence wild-type actin also displays some nuclear localization (Fig. S1), and consequently will interact with nuclear proteins to a certain extent. In line with this, especially the high-confidence interactions obtained with AP-MS for actin, contained a substantial portion (38%) of hits with the GO term nucleus (Fig. 1B). This tendency was most notable with the non-polymerizable actin mutant R62D-actin (Table S1), and can be explained by the molecular properties of R62D-actin: first, it cannot be incorporated into the actin cytoskeleton as tightly as wild-type actin (Posern et al., 2002) and, second, it has a higher nuclear import rate than wild-type actin (Dopie et al., 2012). Hence, the analysis against the wild-type actin without the NLS especially would eliminate those interactions that take place both in the cytoplasm and in the nucleus. This could explain the relatively low number of known actin-binding proteins that were detected as high-confidence interactors for nuclear actin (Fig. 2C). Recent studies have finally shown that nuclear actin can polymerize into canonical actin filaments (Grosse and Vartiainen, 2013), raising the question on how polymerization influences nuclear actin interactions. Although answering this question was not an aim of this study, we utilized the non-polymerizable actin mutant R62D-actin alongside wild-type actin in our MS screen. However, we did not observe major differences between their nuclear interactomes (Fig. 1C). Indeed, most of the known actin interaction partners in the nucleus, including MRTF-A (Vartiainen et al., 2007), pTEF-β (Qi et al., 2011) and hnRNPU (Kukalev et al., 2005), as well as the different chromatin-remodeling complexes (Kapoor et al., 2013; Zhao et al., 1998) seem to bind monomeric actin. It must also be noted that our experimental set up was not geared towards identifying binding partners for nuclear filamentous actin. It is unlikely that the filaments would stay intact during the AP-MS procedure, and the long biotinylation time required in BioID would not be compatible with the transient nature of the nuclear actin filaments (Baarlink et al., 2017, 2013; Plessner et al., 2015). Nevertheless, this is an important avenue for future studies, since nuclear actin polymerization seems to have a key role both during the cell cycle (Baarlink et al., 2017) and in the DNA damage response (Caridi et al., 2018; Schrank et al., 2018).

Beyond its roles in chromatin-remodeling complexes, actin has also been linked to different steps of transcription (Fomproix and Percipalle, 2004; Hofmann et al., 2004; Kukalev et al., 2005; Obrdlik et al., 2008; Percipalle et al., 2003), and our recent genome-wide analysis indicates that actin interacts with basically all transcribed genes (Sokolova et al., 2018). In support of this, multiple proteins related to transcription from the assembly of the PIC to Pol II-mediated elongation (Jonkers and Lis, 2015) were identified as high-confidence interactions in our MS screens (Fig. 3A,B, Table S2). Notably, almost all of these transcription-related hits were obtained with BioID (Fig. 3A,B, Table S2), suggesting that these interactions may be rather transient and dynamic. Alternatively, the interactions could be very context specific, and thus not retained in the AP-MS. Unexpectedly, we did not obtain a single subunit of the core RNA polymerases as a high-confidence interactor in our assays (Table S2), despite the fact actin had already been shown to co-purify with Pol II decades ago (Egly et al., 1984). Whether this is due to the experimental set up, for example the usage of tagged actin, awaits further studies. Beyond gene expression, recent studies have also linked actin to maintenance of genomic integrity via movement of damaged DNA (Caridi et al., 2018; Schrank et al., 2018) and DNA replication (Parisis et al., 2017). Even though our MS analysis was performed in untreated, cycling cells, few of the putative interaction partners for actin were related to DNA replication and the DNA damage response (Fig. 3A, Table S2). In the future, this nuclear actin interactome analysis could be expanded to different cell cycle phases and to samples with induced DNA damage.

It is well established that actin associates with nuclear Arps to bind chromatin remodelers and modifiers like SWI/SNF, SRCAP/Tip60 and INO80 (reviewed in Kapoor and Shen, 2014). To our surprise, the shared hits from the AP-MS and BioID screens included several subunits from the hATAC, a histone-modifying complex without nuclear Arps (Guelman et al., 2009), and our further analysis revealed direct interaction between the histone acetyl transferase KAT14 and actin (Fig. 5E). We found that actin interacts with the C-terminus of KAT14, which also contains the HAT activity, and actin inhibited the KAT14 HAT activity *in vitro* (Figs 5D and 6A,B). The hATAC contains also another HAT, GCN5, and it seems that the HAT activities of both GCN5 and KAT14 contribute to the total HAT activity of the ATAC complex (Guelman et al., 2009; Suganuma et al., 2008). However, KAT14 and GCN5 seem to have distinct histone substrate preferences (Suganuma et al., 2008). In the future, it will be interesting to study whether actin regulates the activity of also the whole hATAC complex, or whether its functions are restricted to KAT14. We were also able to detect significant decrease in H4K5Ac levels, which have previously been shown to be dependent on KAT14 (Guelman et al., 2009), in cells overexpressing either NLS-actin or NLS-R62D-actin (Fig. 6C,D). However, we cannot exclude the possibility that these cellular effects are due to the action of actin on other proteins that control histone acetylation. Indeed, a recent paper demonstrated that actin monomers interact with the histone deacetylases HDAC1 and HDAC2, and that polymerizing nuclear actin increased HDAC activity and decreased histone acetylation; however, the interaction between actin and HDAC proteins did not seem to be direct (Serebryannyy et al., 2016a). Further investigations are needed to reveal how actin is involved in controlling the delicate histone acetylation balance in cells. Interestingly, KAT14 also promotes expression of smooth muscle cell-specific genes by interacting with SRF, CRP2 and myocardin (Ma et al., 2017). It would be interesting to investigate whether KAT14 also plays a role in controlling the actin-regulated myocardin family member MRTF-A (Vartiainen et al., 2007).

Intriguingly, our nuclear actin BioID screen (Fig. 3A,B) identified numerous hits related to pre-mRNA splicing and polyadenylation, including several components of the individual snRNPs, and also protein complexes functioning at the late stages of spliceosome assembly (Prp19 and BIC complexes) (Wahl and Lührmann, 2015). We have previously shown that depletion of certain splicing related factors, such as CDC73, results in the formation of nuclear actin filaments, or bars (Rohn et al., 2011). It is therefore tempting to speculate that actin and RNA splicing are connected and that they both influence each other. In the BiFC assay, the Prp19 complex subunit CDC5L displayed a punctate interaction pattern with actin in the nucleus (Fig. 7A), in addition to being detected in larger nuclear structures, possibly nucleoli. This localization pattern suggests that particularly the interactions involving late spliceosome assembly stages may occur in nuclear speckles, domains known to be enriched in pre-mRNA splicing factors and involved in co-transcriptional splicing (Galganski et al., 2017; Herzel et al., 2017).

Our pre-mRNA splicing minigene assays (Fig. 7B,C) provide a tentative support for an interaction between actin and pre-mRNA splicing machinery. Specifically, we used a set of splicing reporters displaying progressively increasing exon skipping levels (Fig. 7B,C) and found that reporters showing highest level of exon skipping responded to the manipulation of nuclear actin levels seen upon Ipo9 and Exp6 depletion. Significantly, the inclusion of the skipped exons improved with the SMN2 and RPGR −2C reporters, which both showed the lowest initial levels of exon inclusion (Fig. 7C). This could result from either a direct interaction between actin and the pre-mRNA splicing machinery, or the effect could be indirect, for example resulting from a differential expression of spliceosome components or splicing factors. However, we favor yet another, more kinetic, explanation whereby actin influences Pol II elongation or pausing rate. Extensive previous work has established that the bulk of cellular pre-mRNA splicing takes place co-transcriptionally, but has also shown that the elongation and pausing rates of Pol II can influence alternative splicing decisions (Herzel et al., 2017). Specifically, our data is consistent with the previous observation that slower rates of Pol II elongation extends the ‘window of opportunity’ for weak splice sites that are transcribed prior to the competing strong splice site (Fong et al., 2014). We thus hypothesize that the increased alternative exon inclusion seen in the SMN2 and RPGR −2C minigenes after altering nuclear actin levels (Fig. 7C) can be explained by a reduced overall rate of Pol II elongation, which provides more time for the weak splice sites to commit to splicing pathway. This result is consistent with our earlier observation that both Ipo9 and Exp6 depletion lead to decreased transcription (Dopie et al., 2012). Further experiments, such as deep RNA sequencing, are required to elucidate the exact role of actin in transcription versus RNA processing, and to confirm our hypothesis that actin affects the Pol II elongation rate. The BioID hits that link actin to, for example, p-TEFb regulation (Fig. 3B) form an excellent starting point for these studies.

Taken together, our interactome analysis opens new avenues for studying the mechanisms by which actin operates in the nucleus by providing both new angles on known actin-regulated nuclear processes and identifying completely novel interactions as well as functions. The next step will be to analyze these interactions, preferably in biochemical detail. This may prove challenging, since many interactions of actin, for example in the context of transcription, may be highly dynamic and transient, or could be highly context specific, making it difficult to reconstitute these *in vitro*. However, biochemical data for nuclear actin interactions is crucial so that we can start to manipulate the interactions and functions separately. This is critical, because this interactome analysis has further highlighted the multifunctional nature of nuclear actin.

## MATERIALS AND METHODS

### Plasmids

Detailed information of the cloned plasmids are available upon request. The majority of the plasmids generated here are available at Addgene and the Addgene ID number is indicated after the plasmid.

To generate AP-MS compatible Strep-HA–actin constructs, we used the Gateway (GW) compatible N-terminally tagged destination vector pcDNA5/FRT/TO/SH/GW (Glatter et al., 2009) and human β-actin constructs [pENTR-actin (118378), pENTR-NLS-actin (118380), pENTR-R62D-actin (118379) and pENTR-NLS-R62D-actin (118381)]. To generate the BioID compatible N-terminally tagged HA-BirA* destination vector (HA-BirA*-pDEST-N-pcDNA5/FRT/TO, 118375), pcDNA5/FRT/TO/SH/GW was altered by replacing the Strep tag with BirA* from pcDNA 3.1 Myc-BirA (Roux et al., 2012) via restriction digestion. GW compatible vectors used in bimolecular fluorescence complementation (BiFC) assay [HA-GFP1-10-actin-pcDNA4/TO (118370) and Flag-GFP11-pDEST] were cloned from pmGFP1-10 and pmGFP11 plasmids (Brunello et al., 2016), which were kind a gift from Henri Huttunen (Neuroscience Center, University of Helsinki, Finland). GW cloning compatible destination vectors containing N- or C-terminal tags [2HA-pDEST-N/C (118373, 118374), 2Flag-pDEST-N/C (118371, 118372), Flag-GFP11-pDEST-N/C (118366, 118367), HA-GFP1-10-pDEST-C/N (118368, 118369)] were generated by restriction digestion cloning into pDEST27 vector (Thermo Fisher Scientific) as backbone. AP-MS or BioID hits were cloned into Flag-GFP11-pDEST by using GW cloning and entry vectors from the Human ORFeome collection (www.biocenter.helsinki.fi/bi/gbu/orf/), except BRG1/SMARCA5, which was cloned from HeLa cell cDNA. KAT14 was also cloned with GW into 2Flag-pDEST and to pDEST10 (KAT14-pDEST10, 118382) (Thermo Fisher Scientific). KAT14 truncations [pENTR-KAT14-1-546 (118377), pENTR-KAT14-547-782] were cloned into 2Flag-pDEST vector with GW cloning. PENTR-G168D,Y169D-actin (118384) and pENTR-V159N-actin (118383) plasmids were generated from the pENTR-actin plasmid by site mutagenesis and cloned into 2HA-pDEST vector with GW cloning. GFP-actin plasmid has been described in (Dopie et al., 2012). SMN1 and SMN2 minigene plasmids (Roca and Krainer, 2009) as well as RPGR minigene plasmids (Roca et al., 2012) were a kind gift from Xavier Roca (School of Biological Sciences, Division of Molecular Genetics and Cell Biology, Nanyang Technological University, Singapore).

### Antibodies

Antibodies against the following proteins were from Merck: Flag (F1804, 1:400), HA (H3663, 1:400), tubulin (B512, 1:2500) and actin (A3853, 1:2000). Antibodies against the following proteins were from Abcam: KAT14 (ab127040, 1:1000) and H4K5Ac (ab51997, 1:10,000). The antibody against the following protein was from Santa Cruz Biotechnology: HA-Probe (sc-805, 1:300). The antibody against the following protein was from Abnova: Ipo9 (PAB0154, 1:1000). The following secondary antibodies were from Thermo Fisher Scientific: horseradish peroxidase (HRP)-conjugated anti-mouse-IgG (G-21040) (1:7500), HRP-conjugated anti-rabbit-IgG (G-21234, 1:7500), Alexa-Fluor-488-conjugated streptavidin (S11223, 1:500), Alexa-Fluor-594-conjugated anti-mouse-IgG (A11005, 1:500), Alexa-Fluor-594-conjugated anti-rabbit-IgG (A11012, 1:500), Alexa-Fluor-647-conjugated anti-mouse-IgG (A-31571, 1:500). The following secondary antibodies were from Merck: HRP-conjugated Flag M2 (A8592, 1:7500), HRP-conjugated HA (H6533, 1:7500), and HRP-conjugated HIS (A7058, 1:7500).

### Cell lines and transfections

All cell lines [HeLa cells from Marikki Laiho (Institute of Biotechnology, University of Helsinki, Finland), Flp-In™ T-REx™ 293; from Markku Varjosalo (Institute of Biotechnology, University of Helsinki, Finland); U2OS from Pekka Lappalainen (Institute of Biotechnology, University of Helsinki, Finland) and NIH3T3 from Richard Treisman (Signalling and Transcription Laboratory, The Francis Crick Institute, UK)] were cultured in Dulbecco's modified Eagle's medium (DMEM; Merck) supplemented with 10% fetal bovine serum (FBS) (Thermo Fisher Scientific) and penicillin-streptomycin (Thermo Fisher Scientific) and were maintained at 37°C and 5% CO_2_.

For generation of the stable cell lines with inducible expression of the BirA*- and Strep-HA-tagged versions of the different actin constructs (actin, R62D-actin, NLS-actin and NLS-R62D-actin), human embryonic kidney (Flp-In™ T-REx™ 293, HEK) cells (Invitrogen, Life Technologies, R78007, cultured according manufacturer's recommended conditions) were co-transfected with the expression vector and the pOG44 vector (Invitrogen) using the Fugene6 transfection reagent (Roche Applied Science). At 2 days after transfection, cells were selected in 15 μg/ml blasticidin S (Invivogen) and 50 μg/ml hygromycin (Thermo Fisher Scientific) for 2 weeks; then, positive clones were tested, and clones with correct actin localization and strong expression levels were amplified and used for experiments. Stable cells expressing BirA*- or Strep-HA tag fused to green fluorescent protein (GFP) were used as negative controls and processed in parallel to the bait proteins.

The stable cell line used in BiFC assay was generated by transfecting HA-GFP1-10-actin-pcDNA4/TO plasmid into human osteosarcoma (U2OS) cells stably expressing tetracycline repressor by using Lipofectamine 2000 (Thermo Fisher Scientific) according to the manufacturer's protocol. Cells were selected with 300 µg/ml zeocin (Invivogen) and 2.5 µg/ml blasticidin S, and the clones were screened for strong induction level and low background signal. For the BiFC assay, HA–GFP-1-10–actin U2OS cells were plated onto 24-well tissue culture plate at a density of 12,000 cells per well. The next day, cells were induced with 1:1000 dilution of tetracycline. On day three, cells were transfected with DNA constructs using JetPrime transfection reagent (Polyplus transfection), according to the manufacturer's instructions. In total, 250 ng of DNA was used for every transfection reaction, with the experimental vector amount 25 ng. The pEF-vector was used to equalize the total amount of DNA.

### Immunoprecipitations

For immunoprecipitation (IP), HeLa cells were plated onto 10 cm culture dishes at 10^6^ cells per dish. The next day, cells were transfected with 2Flag–KAT14, 2Flag–KAT14 truncations and 2HA–actin constructs (R62D-actin, V159N-actin and G168D,Y169D-actin) using JetPrime transfection reagent (Polyplus transfection), according to the manufacturer's instructions. Equal amount (3 µg) of expression vectors was used for transfection. At 2 days after transfection, cells were collected and lysed in lysis buffer [50 mM Tris-HCl pH 8, 100 mM NaCl, 1% IGEPAL and protease inhibitors (Roche)]. Cleared lysate was obtained by centrifugation and diluted 1:2 in IP buffer [50 mM Tris-HCl, pH 8, 100 mM NaCl, and protease inhibitors (Roche)]. Prewashed anti-Flag M2 affinity gel (Merck) (30 µl) was added to diluted lysates and, after 3 h of rotation at +4°C, the samples were washed three times with IP buffer, and bound proteins eluted with 90 μl of 1× SDS-PAGE loading buffer. Samples (33% of the immunoprecipitates and 10% of the inputs) were separated in 10% SDS-PAGE gels, transferred onto nitrocellulose membrane and the proteins were detected with directly conjugated anti-Flag M2-peroxidase, anti-HA HA-7 peroxidase, or with mouse anti-actin antibodies. Error bars show the s.d. of three independent experiments. Significance between indicated samples is indicated by an asterisk (*P<0.05).

### MS sample preparation and MS analysis

Strep-HA or HA-BirA* stable HEK cell lines were expanded to 80% confluence in 6×150 mm cell culture plates. For the AP-MS approach, 1 μg/ml tetracycline was added for 24 h for induction of protein expression before harvesting the cells. For BioID, in addition to tetracycline, 50 μM biotin (Thermo Fisher Scientific) was added 24 h before harvesting. Cells from confluent plates (∼6×10^7^ cells) were pelleted as one biological sample, and at least two biological replicates were prepared for each bait protein. Cell pellets were snap frozen and stored at −80°C until sample preparation.

For AP-MS, the cell pellet was lysed in 3 ml of lysis buffer 1 (1% IGEPAL, 50 mM Hepes, pH 8.0, 150 mM NaCl, 50 mM NaF, 5 mM EDTA, supplemented with 0.5 mM PMSF and protease inhibitors; Roche). For BioID, the cell pellet was lysed in 3 ml lysis buffer 2 (1% IGEPAL, 50 mM Hepes, pH 8.0, 150 mM NaCl, 50 mM NaF, 5 mM EDTA, 0.1% SDS, supplemented with 0.5 mM PMSF and protease inhibitors; Roche). Lysates were sonicated and treated with benzonase (Merck). Cleared lysate was obtained by centrifuging the crude lysate two times, first for 16,000 ***g*** for 15 min and second for at 16,000 ***g*** for 10 min, and loaded consecutively on spin columns (Bio-Rad) containing lysis buffer 1 prewashed 200 μl Strep-Tactin beads (IBA, GmbH). The beads were then washed three times with 1 ml of lysis buffer 1 and four times with 1 ml of wash buffer (50 mM Tris-HCl, pH 8.0, 150 mM NaCl, 50 mM NaF, 5 mM EDTA). Following the final wash, beads were then resuspended twice in 300 μl elution buffer (50 mM Tris-HCl, pH 8.0, 150 mM NaCl, 50 mM NaF, 5 mM EDTA, 1 mM Biotin) for 5 min each time and eluates were collected into Eppendorf tubes. Samples were snap frozen and stored at −20°C until MS-LC analysis.

MS-liquid chromatography (LC) samples were treated with 5 mM Tris (2-carboxyethyl)phosphine (TCEP) and 10 mM iodoacetamide for 30 min at 37°C to reduce cysteine bonds and alkylation, respectively. The proteins were then digested to peptides with sequencing grade modified trypsin (Promega, V5113) at 37°C overnight. After quenching with 10% trifluoroacetic acid (TFA), the samples were desalted by using C18 reversed-phase spin columns according to the manufacturer's instructions (Harvard Apparatus). The eluted peptide sample was dried in a vacuum centrifuge and reconstituted to a final volume of 30 μl in 0.1% TFA and 1% CH_3_CN.

The MS analysis was performed on Orbitrap Elite hybrid mass spectrometer coupled to EASY-nLC II -system using the Xcalibur version 2.7.0 SP1 (Thermo Fisher Scientific); 4 μl of the tryptic peptide mixture was loaded into a C18-packed pre-column (EASY-Column™ 2 cm×100 μm, 5 μm, 120 Å, Thermo Fisher Scientific) in 10 μl volume of buffer A and then to C18-packed analytical column (EASY-Column™ 10 cm×75 μm, 3 μm, 120 Å, Thermo Fisher Scientific). A 60-min linear gradient at the constant flow rate of 300 nl/min from 5% to 35% of buffer B (98% acetonitrile and 0.1% formic acid in MS grade water) was used to separate the peptides. Analysis was performed in data-dependent acquisition: one high resolution (60,000) Fourier transform MS (FTMS) full scan (*m*/*z* 300–1700) was followed by the top 20 collision-induced dissociation (CID) MS2 scans in ion trap (energy 35). The maximum FTMS fill time was 200 ms (full AGC target 1,000,000) and the maximum fill time for the ion trap was 200 ms (MSn AGC target of 50,000). Precursor ions with more than 500 ion counts were allowed for MSn. To enable the high resolution in FTMS, scan preview mode was used.

### Search parameters and acceptance criteria

Proteins were identified using Proteome Discoverer™ software with SEQUEST search engine (version 1.4, Thermo Fisher Scientific). The .raw files were searched against the human component of the UniProt database (release 2014_11; 20130 entries) complemented with trypsin, BSA, GFP and the tag sequences. Trypsin was used as the enzyme specificity. Search parameters specified a precursor ion tolerance of 15 ppm and fragment ion tolerance of 0.8 Da, with up to two missed cleavages allowed for trypsin. Carbamidomethylation (+57.021464 Da) of cysteine residues was used as static modification whereas oxidation (+15.994491 Da) of methionine and biotinylation (+226.078 Da) of lysine residues or N terminus were used as dynamic modifications. The peptide false discovery rate (FDR) was calculated using Percolator node software and set to <0.01. Spectral counting was used to produce semi-quantitative data. Identification metrics for each sample are listed in Table S1.

### Identification of high-confidence interactions

Significance Analysis of INTeractome (SAINT)-express version 3.6.0 (Teo et al., 2014) and Contaminant Repository for Affinity Purification (CRAPome, http://www.crapome.org/) (Mellacheruvu et al., 2013) were used for identification of specific high-confidence interactions (HCI) from our MS data. A total of 11 GFP–Strep-HA runs (one GFP–Strep-HA control was performed by the authors and 10 other control runs were obtained from www.crapome.org and each number indicates a specific CRAPome GFP–Strep-HA control run used for the analysis; CC295, CC306, CC307, CC411, CC316, CC323, CC328, CC329, CC330 and CC332) were used as control counts for each AP-MS hit. Four HA-BirA*-GFP runs (from two biological HA-BirA*-GFP replicates) were used as control counts for each BioID-MS hit. Actin-MS and R62D-actin MS replicates (total four in AP-MS and in BioID-MS) were combined to analyze the AP-MS and BioID actin interactomes. NLS-actin MS and NLS-R62D-actin MS replicates (a total of four in AP-MS and six in BioID-MS) were combined to analyze the nuclear actin AP-MS and BioID interactomes. Identified proteins were considered as high-confidence interactions if they were identified in at least two biological replicates, and had an average spectral count fold change of ≥2 (in AP-MS) or ≥0.5 (in BioID) compared to control. In addition, the HCI for nuclear actin had average spectral count fold change ≥1 (in AP-MS) or ≥0.5 (in BioID) compared to the respective actin sample. For SAINT-express analysis, spectral counts of different hit candidates (two or three biological replicates) were pooled and analyzed over control with SAINT Express. The final results only consider proteins with SAINT score ≥0.88. This corresponds to an estimated protein-level Bayesian FDR of <0.01. Endogenously biotinylated proteins such as acetyl-CoA carboxylase 2 (ACACB) and acetyl-CoA carboxylase 1, (ACACA) as well as proteins from other species than *Homo sapiens* were removed from the final HCI list. Final HCIs are listed in Table S2. Database for Annotation, Visualization and Integrated Discovery (DAVID) annotation tool (Huang et al., 2009) was used to create GO term tables from the hit lists. Shared hits, specific AP-MS hits and KAT14 related hits were visualized with STRING map software (version 10.5) (Szklarczyk et al., 2017).

### Immunofluorescence and microscopy

Cells on coverslips were fixed with 4% paraformaldehyde for 15 min, washed three times with PBS and permeabilized for 7 min with 0.2% Triton X-100 (Merck) in PBS. For antibody staining, permeabilized cells were blocked with blocking buffer [1% gelatin (Merck), 1% BSA (Merck) and 10% FBS (Lonza) in 1× PBS] for 1 h and incubated with primary antibody for 1 h. Coverslips were washed and incubated with Alexa Fluor-conjugated secondary antibody for 1 h. Coverslips were washed three times with 1× PBS, once with MQ water and mounted in Prolong Diamond with DAPI (Thermo Fisher Scientific).

A wide-field fluorescence Leica DM6000 (Leica, Welzlar, Germany) microscope with a HCXPL APO 63×/1.40–0.60 oil objective, and confocal Leica TCS SP8 STED 3X CW 3D microscope with a HC PL APO 93×/1.30 glycerol objective were used to image induced HEK cell lines and BiFC samples. The image files for these images were processed with Leica LAS X and ImageJ software.

Images of the fixed NIH3T3 cells were used to measure the GFP-actin levels in Ipo9- and Exp6-depleted cells were acquired with a confocal Zeiss LSM 700 microscope with a LCI Plan-Neofluar 63×/1.30 Imm Corr glycerol objective and Zeiss ZEN 2 blue edition software. GFP–actin intensities were measured from equal areas from the cell nucleus and cytoplasm by using ImageJ software. The ratio was calculated by diving the average nuclear intensity by the average cytoplasmic intensity.

### Insect cell expression and protein purification

Recombinant human His-KAT14 was produced by using the MultiBac baculovirus expression vector system. Briefly, the KAT14-pDEST10 plasmid was transformed into DH10MultiBac cells and recombinant bacmids were isolated and the presence of the coding sequence of KAT14 was confirmed by PCR. A bacmid containing the correct sequence was transfected into *Spodoptera frugiperda* cells (Sf9) by using Fugene HD (Promega). The baculoviruses were amplified and used for protein expression. The expression of recombinant human KAT14 was conducted by infection 2×106 Sf9 cells with the baculoviral stock at a multiplicity of infection (MOI) of 1. The cells were harvested after 72 h and the cell pellets were snap frozen and stored at −80°C

Cell pellets were resuspended in lysis buffer [25 mM Tris-HCl pH 8.0, 10 mM NaCl, 1 mM MgCl_2_, 2 mM B-mercabtoethanol, 0.2% IGEPAL, 1 mM PMSF, protease inhibitors (Roche)] with Benzonase (Merck) and lysed with EmulsiFlex-C3 (AVESTIN) with a gauge pressure of ∼15,000 psi for 10 min. Immediately after lysing, the NaCl concentration was adjusted to 150 mM and 10 mM Imidazole was also added to cells extracts. Cell extract was incubated for 10 min at +4°C and then clarified by a centrifugation at 92,000 ***g*** for 30 min and immediately processed by metal (Ni^2+^) affinity purification. The cell extract from ∼1 l of infected cells was mixed with Ni^2+^-NTA agarose beads (Qiagen) that were equilibrated with lysis buffer. Beads were incubated with extract for 2 h in rotation at +4°C. The beads were washed three times with the buffer A (25 mM Tris-HCl pH 8.0, 300 mM NaCl, 2 mM mM B-mercaptoethanol) and two times with buffer A with addition of 10 mM Imidazole. The last wash was conducted in a disposable 10 ml Poly-prep column (Bio-RAD) and the protein was eluted from the beads with 500 mM Imidazole in lysis buffer. The protein was further purified with gel filtration column Superdex 200 (Superdex 200 HiLoad 16/60, Pharmacia), which was equilibrated with 25 mM Tris-HCl pH 7.5, 150 mM NaCl, 0.5 mM EDTA, 2 mM B-mercaptoethanol and 10% glycerol. Fractions were analyzed by SDS-PAGE, and fractions containing His–KAT14 were concentrated and stored at −80°C.

### Pulldown with purified proteins

His–GST lysate (500 µl) and purified His–KAT14 were immobilized on Ni^2+^-NTA agarose beads equilibrated with 30 µl storage buffer (25 mM Tris-HCl pH 7.5, 150 mM NaCl, 0.5 mM EDTA, 2 mM B-mercaptoethanol, 10% glycerol; Qiagen) for 2 h on rotation at +4°C. Beads were washed once with binding buffer 1 (25 mM Tris-HCl pH 7.5, 150 mM NaCl and 1 mM DTT), three times with binding buffer 2 (25 mM Tris-HCl pH 7.5, 500 mM NaCl and 1 mM DTT) and once with G-Buffer (5 mM Tris-HCl pH 7.5, 0.2 mM ATP, 0.2 mM CaCl_2_ and 0.5 mM DTT). After the washes, beads were incubated with a 4-fold amount of rabbit skeletal muscle actin compared to His–KAT14 in G-buffer for 3 h with rotation at +4°C. Rabbit skeletal muscle actin was prepared as described in Pardee and Spudich (1982). After incubation the beads were washed three times with G-buffer and bound proteins were eluted with 1×SDS-PAGE loading buffer. Samples were boiled for 5 min and the proteins were separated in 7.5% Mini-PROTEAN^®^ TGX™ Precast Protein Gel (BioRAD) and electroblotted to nitrocellulose membrane. The membrane was probed with anti-KAT14, -actin or -His antibodies to assess their association with His-fusions.

### HAT assays *in vitro* and in cells

Core histones (5 µg; Merck) were incubated with 30 µM acetyl CoA in different conditions: with MQ water, with 20 µg HeLa nuclear extract, with 250 nM His–KAT14, with 250 nM His–KAT14 with addition of 2.5 µM actin and with 2.5 µM actin alone. 25 µl reactions were performed in 1× HAT buffer (50 mM Tris-HCl, pH 8.0, 50 mM NaCl, 0.1 mM EDTA, 0.01% Igepal) and incubated in +30°C for 1 h. After incubation the proteins were denaturated in 1× SDS loading buffer and boiled for 5 min before separation by 7.5% Mini-PROTEAN^®^ TGX™ Precast Protein Gel and electroblotted to nitrocellulose membrane or stained with Coomassie. The membrane was probed with anti-KAT14, -actin and -H4K5Ac antibodies. Relative H4K5Ac levels were normalized to the H4 protein amount and the values for HeLa nuclear extract were set to 1. Error bars show the s.d. of three independent experiments. Significance between indicated samples is indicated by an asterisk (*P<0.05).

Strep-HA–NLS-actin and Strep-HA–NLS-R62D-actin cells were plated in six-well plate wells at 250,000 cells per well. The next day, half of the cells were induced with a 1:1000 dilution of tetracycline. On day three, the all the cells were harvested and washed once with 1× PBS before lysing into 1× SDS loading buffer. Samples were boiled for 5 min and the proteins were separated on a 7.5% Mini-PROTEAN^®^ TGX™ Precast Protein Gel and electroblotted onto nitrocellulose membrane. The membrane was probed with anti-KAT14, -actin, -tubulin and -H4K5Ac antibodies. For quantification, the relative H4K5Ac amount was normalized to tubulin and the values of non-induced NLS-actin or NLS-R62D-actin were set to 1. Error bars show the s.d. of four independent experiments. Significance between indicated samples is indicated by an asterisk (*P<0.05).

### Minigene splicing assay

For the splicing assay, NIH3T3 cells were plated onto six-well tissue culture plate at a density of 40,000 cells per well. The following day, cells were transfected with 20 nmol siRNA (Ipo9: 5′-CACCGAGGAGCAGAUUAAA-3′, Exp6 5′-CGUUGAUAUUGGACGCCAA-3′ from Sigma; negative control: AllStars negative control from Qiagen) using Interferin transfection reagent (Polyplus transfection), according to manufacturer's instructions. On day 4, cells were re-transfected with siRNAs and with the SMN1 and SMN2 minigene plasmids (75 ng of SMN minigene, total DNA amount 1 µg per well added up with mock DNA) [previously described in Roca and Krainer (2009)] or with RPGR minigene plasmids (RPGR wt, RPGR −2C and RPGR +6C) (75 ng of SMN minigene, total DNA amount 1 µg per well added up with mock DNA) [previously described in Roca et al. (2012)] with JetPrime transfection reagent (PolyPlus transfection). The following day, total RNA was extracted with Nucleospin RNA II kit instructed by the manufacturer (Macherey-Nagel). Total RNA (500 ng) was used for cDNA synthesis using a Maxima First Strand cDNA Synthesis Kit (Thermo Fisher Scientific). cDNAs from the SMN1/2 minigenes were amplified with pCI-Fwb and pCI-Rev primers (Cartegni et al., 2006) with Phusion Hot Start (Thermo Fisher Scientific) over 25 cycles. cDNAs from the RPGR minigenes were amplified with pcDNA-F and pcDNA-R primers (Roca et al., 2012) with Phusion Hot Start (Thermo Fisher Scientific) over 24 cycles. PCR products were separated on 6 or 8% native PAGE gels and stained with 0.5 µg/ml ethidium bromide. Gel images were acquired with a Gel Doc™ EZ system (BioRAD) and the intensity of the bands corresponding to different splice variants were measured. In all cases, the standard deviations were ≤5%, such that the exon-inclusion percentage values can be compared between experiments. Significance between control siRNA and indicated samples are indicated by an asterisk (*P<0.05).

### Real-time quantitative PCR

Ipo9 and Exp6 depletions were determined via qPCR from the RNA obtained from splicing assay. Briefly, 500 ng of total RNA was used for cDNA synthesis using the Maxima First Strand cDNA synthesis kit (ThermoFisher Scientific). qPCR was carried out using the Bio-Rad CFX machine (Bio-Rad) and SYBR green qPCR reagent (ThermoFisher Scientific). Gene specific primers are as follows: Ipo9_for, 5′-GGAGGTGACAGAGGAATTTGG-3′, Ipo9_rev, 5′-CTCTGATTGGGCACACCAGT-3′; Exp6_for, 5′-TTCCTCCCCAGCATTATCGC-3′; Exp6_rev, 5′-GCGAGGACAGTGGACTTGAA-3′; Gapdh_for, 5′-TGCACCACCAACTGCTTAGC-3′; and Gapdh_rev: 5′-GGCATGGACTGTGGTCATGAG-3′. Relative expression levels were calculated by the comparative CT method, normalizing to the *Gapdh* cDNA: 2−CT (target)/2−CT (Gapdh).

### Statistical analyses

Statistical analyses were performed in Excel. The data for the HAT assays from the western blots and the splicing assays from native PAGE were analyzed with a two-tailed Student's *t*-test because the data conformed to a normal distribution. The data for Strep-HA–NLS-actin and Strep-HA–R62D-NLS-actin cell induction assays was analyzed with a one-sided Student's *t*-test. The data for co-IP assays from the western blots was analyzed with a one-sided Student's *t*-test. Significance was set as *P*<0.05, and the actual *P-*values are indicated in the figure legends. Uncropped images of used blots and native PAGE gels are shown in Figs S6 and S7.

## Supplementary Material

Supplementary information
