# Supplementary material for: Nuclear actin interactome analysis links actin to KAT14 histone acetyl transferase and mRNA splicing
Source: J Cell Sci. 2019 Apr 17;132(8):jcs226852. doi: 10.1242/jcs.226852 (PMC6503952; doi:10.1242/jcs.226852)
Supplement: Supplementary information [file joces-132-226852-s1.pdf]

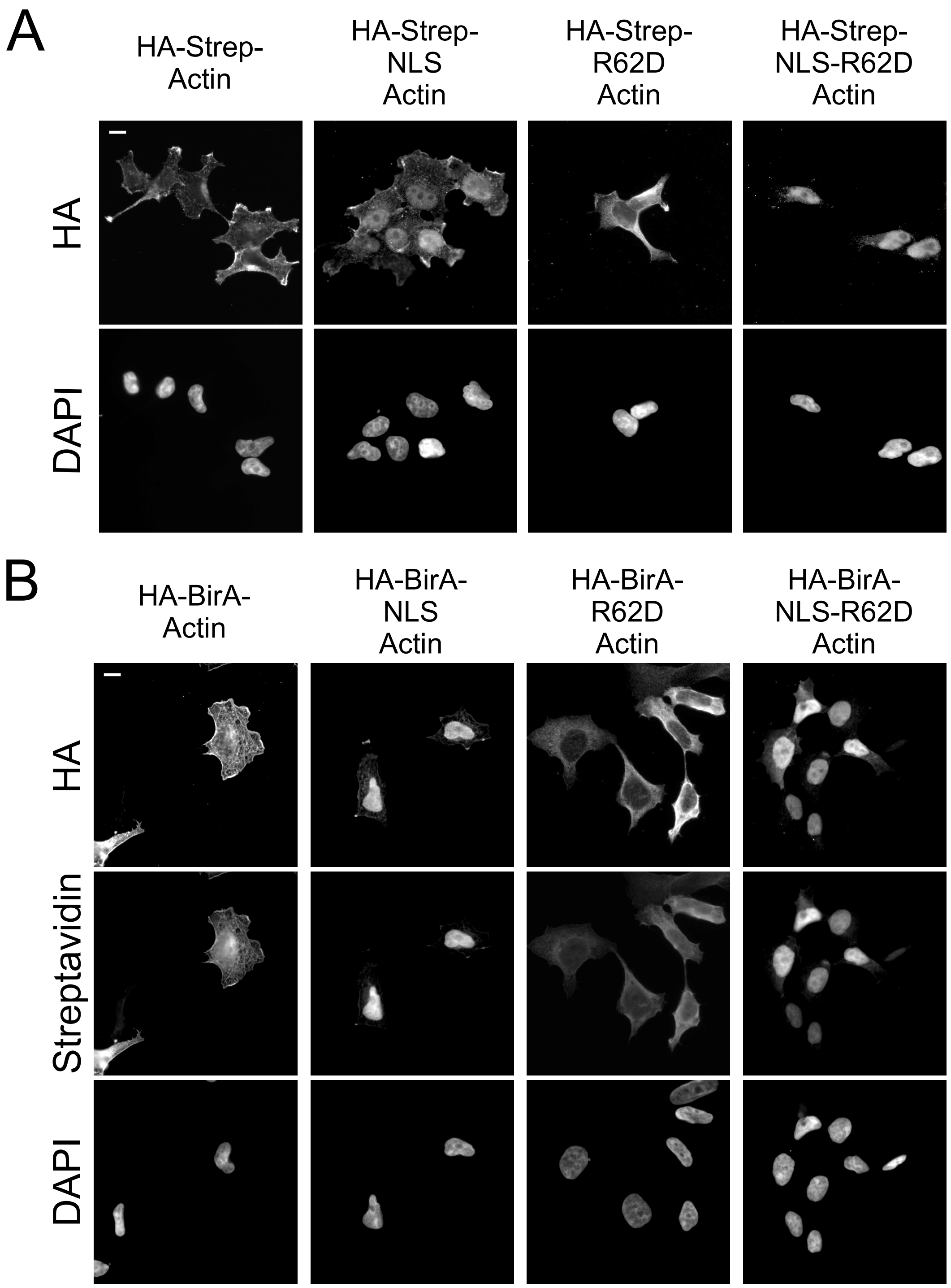

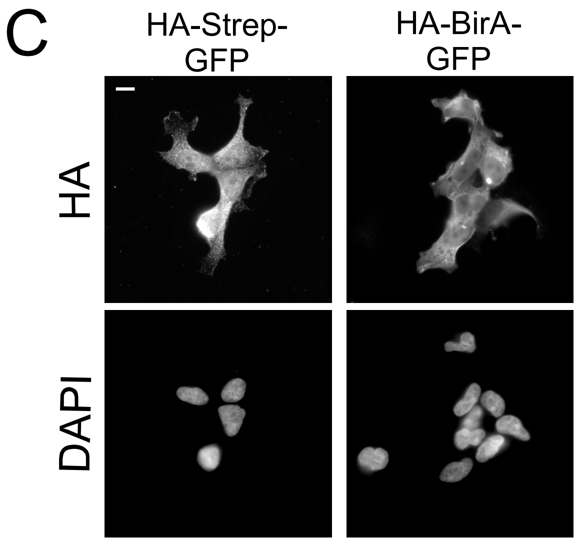

Figure S1. Stable cell lines used in MS experiments
A. Wide-field microscopy images of stable, inducible HEK Flp-In cell lines expressing HA-Strep-tagged actin constructs as indicated, and stained with HA antibody and DAPI. Scale bar, 10 µm.
B. Wide-field microscopy images of stable, inducible HEK Flp-In cell lines expressing HA-BirA-tagged actin constructs as indicated, and stained with HA antibody for construct expression, fluorescent streptavidin to visualize the biotinylated proteins and DAPI. Scale bar, 10 µm.
C. Wide-field microscopy images of stable, inducible HEK Flp-In cell lines expressing Strep-HA or BirA*-tagged GFP used as controls in the MS analysis, stained with HA antibody and DAPI. Scale bar 10 µm.

Journal of Cell Science • Supplementary information

A

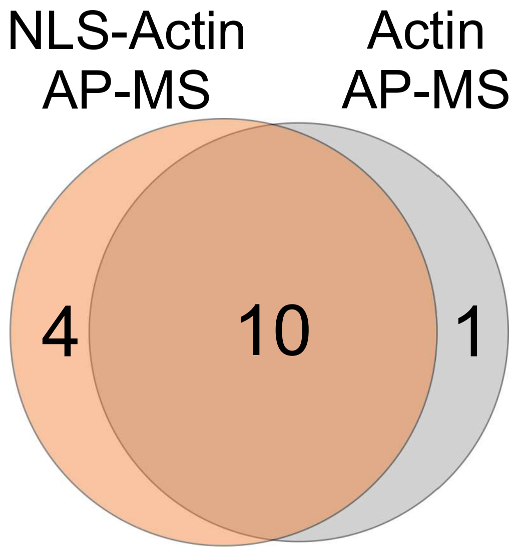

B

GO number	Term	%	PValue	Benjamini
GO:0098609	cell-cell adhesion	16	7.67E-11	6.67E-08
GO:0006357	regulation of transcription from RNA polymerase II promoter	13	1.34E-05	8.30E-04
GO:0006413	translational initiation	12	6.05E-10	1.31E-07
GO:0006364	rRNA processing	12	6.49E-08	6.27E-06
GO:0019083	viral transcription	11	1.34E-09	2.33E-07
GO:0000184	nuclear-transcribed mRNA catabolic process, nonsense-mediated decay	11	2.43E-09	3.53E-07
GO:0006412	translation	11	2.91E-06	2.11E-04
GO:0006614	SRP-dependent cotranslational protein targeting to membrane	10	5.00E-09	6.21E-07
GO:0006338	chromatin remodeling	9	4.66E-08	5.07E-06
GO:0043488	regulation of mRNA stability	9	1.92E-07	1.67E-05
GO:0043967	histone H4 acetylation	8	5.39E-10	1.56E-07
GO:0043968	histone H2A acetylation	7	1.95E-10	8.49E-08
GO:0040008	regulation of growth	7	7.44E-07	5.89E-05
GO:0043044	ATP-dependent chromatin remodeling	5	1.00E-05	6.69E-04

C

GO number	Term	%	PValue	Benjamini
GO:0006614	SRP-dependent cotranslational protein targeting to membrane	17	3.72E-10	1.83E-07
GO:0019083	viral transcription	17	1.53E-09	3.75E-07
GO:0000184	nuclear-transcribed mRNA catabolic process, nonsense-mediated decay	17	2.48E-09	4.05E-07
GO:0006413	translational initiation	17	7.57E-09	7.44E-07
GO:0006364	rRNA processing	17	2.43E-07	1.99E-05
GO:0006412	translation	17	8.65E-07	5.31E-05
GO:0098609	cell-cell adhesion	17	1.45E-06	7.91E-05
GO:0006338	chromatin remodeling	15	6.95E-09	8.54E-07
GO:0043044	ATP-dependent chromatin remodeling	10	6.40E-07	4.49E-05
GO:0043968	histone H2A acetylation	8	1.17E-05	5.75E-04
GO:0006337	nucleosome disassembly	8	1.74E-05	7.77E-04

Figure S2. Nuclear terms obtained with actin AP-MS and NLS-actin AP-MS link actin to similar GO terms

A. Number of unique and shared GO-terms of proteins, which were categorized with Cellular Component (CC) GO term nucleus (GO:0005634) and were from actin AP-MS or NLS-actin AP-MS that was filtered only with GFP (and not with actin without the NLS).

B. DAVID functional annotation chart (Cut off Benjamini 10-4) with GO Direct Biological Pathway (BP) from NLS-actin AP-MS hits filtered only with GFP (and not with actin without the NLS), which were categorized with Cellular Component (CC) GO term nucleus (GO:0005634). Colors as in A.

C. DAVID functional annotation chart (Cut off Benjamini 10-4) with GO Direct Biological Pathway (BP) from actin AP-MS HCIs, which were categorized with Cellular Component (CC) GO term nucleus (GO:0005634). Colors as in A.

Journal of Cell Science • Supplementary information

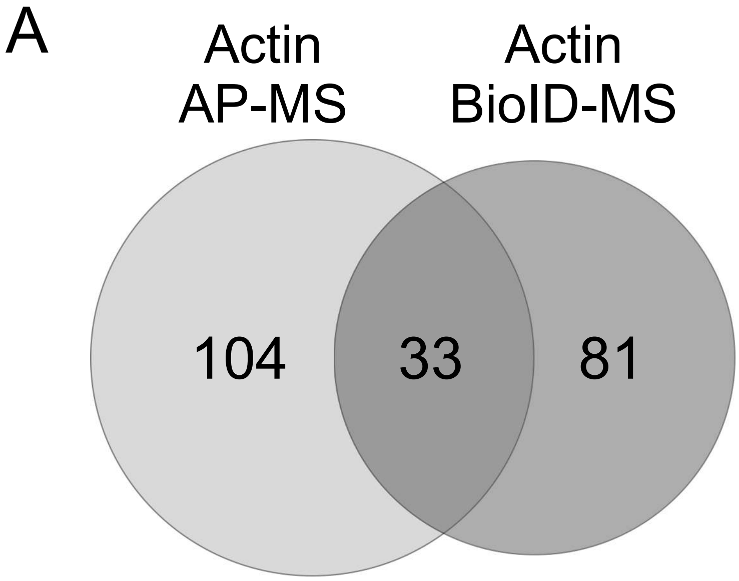

B

GO Number	Term	%	PValue	Benjamini
GO:0005737	cytoplasm	64	3.55E-05	4.22E-04
GO:0005829	cytosol	61	1.22E-07	3.27E-06
GO:0070062	extracellular exosome	45	8.16E-05	7.94E-04
GO:0098609	cell-cell adhesion	30	1.32E-09	1.31E-07
GO:0005913	cell-cell adherens junction	30	2.21E-09	2.36E-07
GO:0005925	focal adhesion	30	1.17E-08	4.16E-07
GO:0030036	actin cytoskeleton organization	27	9.34E-11	1.86E-08
GO:0015629	actin cytoskeleton	27	2.30E-09	1.23E-07
GO:0005856	cytoskeleton	24	2.36E-06	3.61E-05
GO:0030027	lamellipodium	21	2.56E-07	4.57E-06
GO:0005903	brush border	15	3.13E-06	4.18E-05
GO:0005938	cell cortex	15	5.40E-05	5.78E-04
GO:0005869	dynactin complex	12	2.48E-07	5.31E-06
GO:0008154	actin polymerization or depolymerization	12	1.37E-06	9.07E-05

Figure S3. HCIs from the actin AP-MS and actin BioID link actin to cytoskeleton and cell movement
A. Number of unique and shared high confidence interactions from AP-MS and BioID screen for actin.
B. DAVID functional annotation chart (Cut off Benjamini 10-4) with GO Direct Biological Pathway (BP), Cellular Component (CC), Molecular Function (MF) from shared high confidence interactions from actin AP-MS and BioID screens hits.

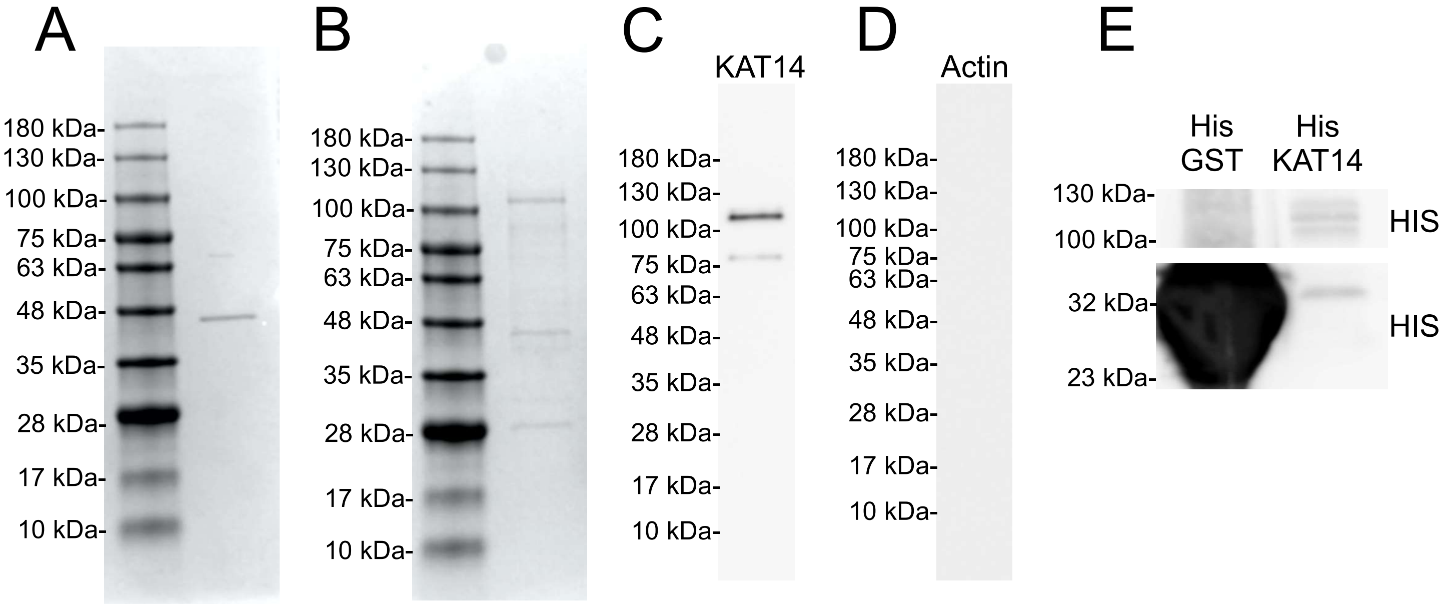

Figure S4. Purified proteins used in the study: actin and KAT14
A. Coomassie stained SDS-PAGE-gel with purified actin (assumed molecular weight ~42 kDa)
B. Coomassie stained SDS-PAGE-gel with purified KAT14 (assumed molecular weight ~105 kDa).
C. Purified KAT14 stained with KAT14 antibody
D. Purified KAT14 stained with actin antibody
E. Western blots of equal amounts of samples (33 %) loaded from the NTA Ni2+ pull down assay with His tagged GST and KAT14. HIS-HRP antibody was used to detect bound proteins in western blot. Related to figure 5E.

Journal of Cell Science • Supplementary information

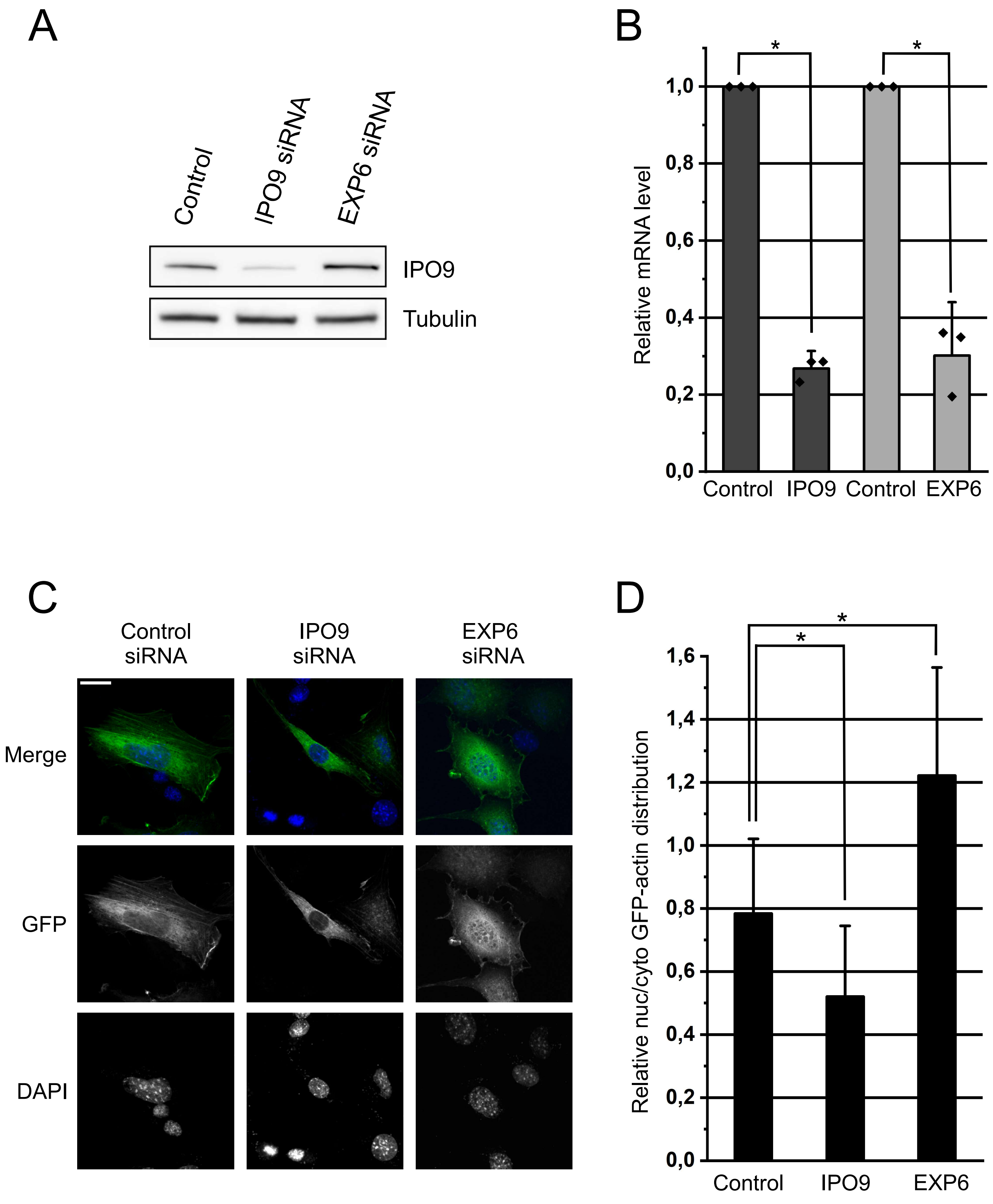
A
Control
IPO9 siRNA
EXP6 siRNA
IPO9
Tubulin
B
Relative mRNA level
1,0
0,8
0,6
0,4
0,2
0,0
*
*
Control
IPO9
Control
EXP6
C
Control siRNA
IPO9 siRNA
EXP6 siRNA
Merge
GFP
DAPI
D
Relative nuc/cyto GFP-actin distribution
1,6
1,4
1,2
1,0
0,8
0,6
0,4
0,2
0,0
*
*
Control
IPO9
EXP6

Figure S5. Depletion of Ipo9 and Exp6 alter nuclear actin levels

A. Western blots from total cell lysates NIH3T3 cells, which have been treated with indicated siRNAs (Control, Ipo9 or Exp6) and stained with the indicated antibodies.

B. Analysis of Ipo9 and Exp6 expression in cells transfected with indicated siRNAs. Expression levels were measured with quantitative reverse transcription-PCR using ΔΔCt method. Values were normalized to Gapdh. Quantification from three independent experiments, error bars are S.D. Dots in the graph represent individual data points from the independent experiments. Statistics with one sided student's t-test show significance between indicated samples Control vs. Ipo9 (P=5.82E-4) and Control vs. Exp6 (P= 0.006) and this is indicated with asterisk.

C. Confocal microscopy images of GFP-actin transfected NIH3T3 cells, which are treated with indicated siRNAs (Control, Ipo9 or Exp6). Scale bar, 20 µm

D. Relative ratio of nuclear (nuc) and cytoplasmic (cyt) GFP-actin fluorescence intensities quantified with ImageJ. Data represent mean fluorescent intensity signal ratios from 15 cells per condition with error bars S.D. Statistics with two sided student's t-test show significance between indicated samples Control vs. Ipo9 (P=6.35E-5) and Control vs. Exp6 (P= 2.23E-6) and this is indicated with asterisk.

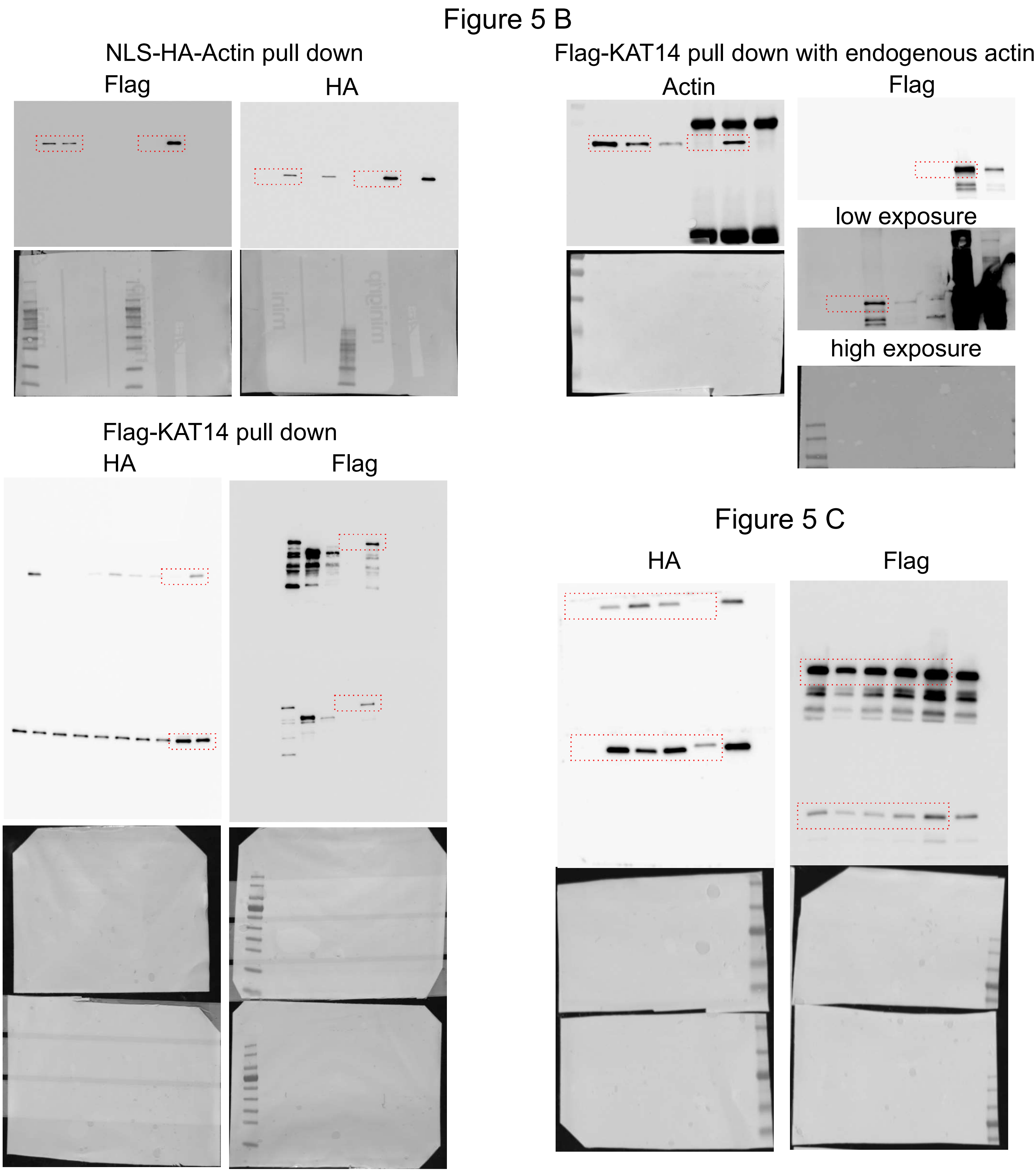

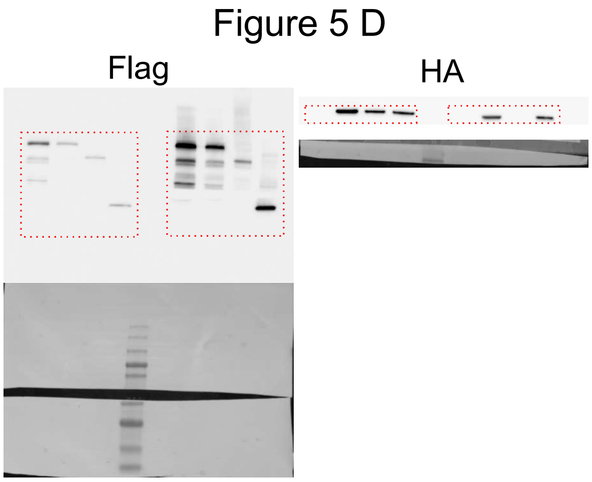

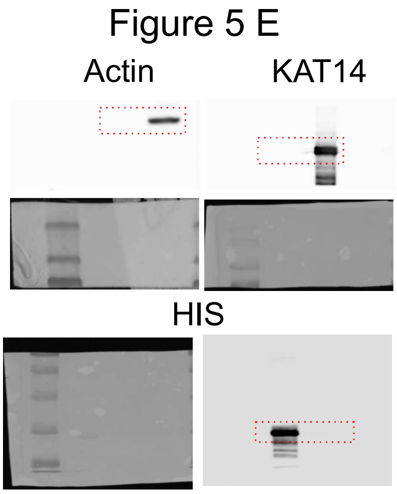

Figure S6. Full western blots used in the main figure 5
Full images of the western blots, which have been used in making the main figure 5, each panel is indicated above the blot. Cropped area, which is shown in the main figures, is indicated in the full blot with red dotted rectangle.

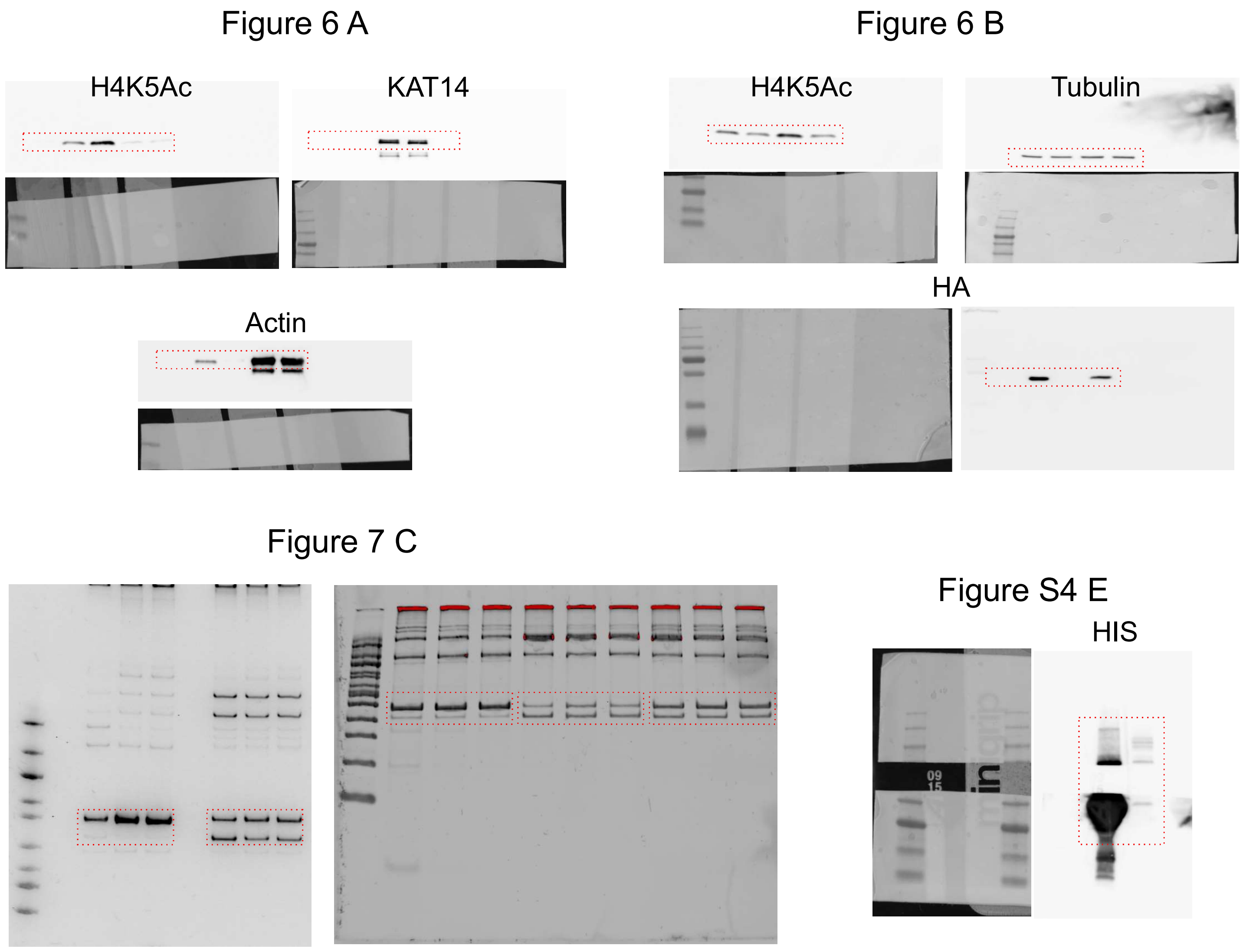

Figure S7. Full western blots and native PAGE gels used in the main figures 6, 7 and figure S4
Full images of the western blots and native PAGE gels, which have been used in making of the main figures (indicated above each blot). Cropped area, which is shown in the figures, is indicated in the full blot with red dotted rectangle.

Table S1. Raw data from the MS analysis
All the MS samples used in the study. The first set of numbers represent the specific MS-RUN and the name tells the origin of the sample. Samples are marked by BirA (BioID) or STREP (AP-MS) to indicate the MS approach used with the sample. Different cell lines are indicated by actin, R62D-actin, NLS-actin or NLS-R62D

Click here to Download Table S1

Table S2. (Related to Fig. 1-3) High-confidence interactions (HCIs) obtained with BioID and AP-MS techniques.
HCIs of all the interactomes (NLS-actin AP-MS, NLS-actin BioID, actin AP-MS, actin BioID) in this study. Interactions are assigned as HCIs based on their average spectral count fold change ≥ 2 (in AP-MS) or ≥ 0.5 (in BioID) compared to control and their statistical filtering score (SAINT probability of cut-off ≥0.88) from the pooled samples. The nuclear actin interactome HCIs also had average spectral count fold change ≥ 1 (in AP-MS) or ≥ 0.5 (in BioID) compared to average spectral count from WT-actin AP-MS or WT-actin BioID. Shared hits between AP-MS and BioID interactomes are indicated with red.
Last page: AP-MS unique; DAVID functional annotation chart (Cut off Benjamini 10^-3) with GO Direct Biological Pathways from unique AP-MS hits.

Click here to Download Table S2
